# Small-molecule targeting of MUSASHI RNA-binding activity in acute myeloid leukemia

**DOI:** 10.1038/s41467-019-10523-3

**Published:** 2019-06-19

**Authors:** Gerard Minuesa, Steven K. Albanese, Wei Xie, Yaniv Kazansky, Daniel Worroll, Arthur Chow, Alexandra Schurer, Sun-Mi Park, Christina Z. Rotsides, James Taggart, Andrea Rizzi, Levi N. Naden, Timothy Chou, Saroj Gourkanti, Daniel Cappel, Maria C. Passarelli, Lauren Fairchild, Carolina Adura, J. Fraser Glickman, Jessica Schulman, Christopher Famulare, Minal Patel, Joseph K. Eibl, Gregory M. Ross, Shibani Bhattacharya, Derek S. Tan, Christina S. Leslie, Thijs Beuming, Dinshaw J. Patel, Yehuda Goldgur, John D. Chodera, Michael G. Kharas

**Affiliations:** 1 Molecular Pharmacology Program, Experimental Therapeutics Center and Center for Stem Cell Biology, Memorial Sloan-Kettering Cancer Center, New York, NY 10065 USA; 2 Louis V. Gerstner Jr. Graduate School of Biomedical Sciences, Memorial Sloan Kettering Cancer Center, New York, NY 10065 USA; 3 Computational and Systems Biology Program, Sloan Kettering Institute, Memorial Sloan Kettering Cancer Center, New York, NY 10065 USA; 4 Structural Biology Program, Sloan Kettering Institute, Memorial Sloan Kettering Cancer Center, New York, NY 10065 USA; 5 Weill Cornell Medical College, Tri-Institutional MD-PhD Program, Rockefeller University and Sloan Kettering Institute, New York, NY 10065 USA; 6 Department of Pharmacology, Weill Cornell Graduate School of Medical Sciences, New York, NY 10065 USA; 7 Chemical Biology Program, Sloan Kettering Institute and Tri-Institutional Research Program, Memorial Sloan Kettering Cancer Center, 1275 York Avenue, New York, NY 10065 USA; 8 Tri-Institutional Training Program in Computational Biology and Medicine, Weill Cornell Medical College, New York, NY 10065 USA; 9 Schrödinger GmbH, Q7, 23, 68161 Mannheim, Germany; 10 High-Throughput and Spectroscopy Resource Center, The Rockefeller University, New York, NY 10065 USA; 11 Hematologic Oncology Tissue Bank, Department of Medicine, Memorial Sloan Kettering Cancer Center, New York, NY 10065 USA; 12 Center for Hematologic Malignancies, Memorial Sloan Kettering Cancer Center, New York, NY 10065 USA; 13 Northern Ontario School of Medicine, Sudbury, ON P3E 2C6 Canada; 14 New York Structural Biology Center, NMR Group, New York, NY 10027 USA; 15 Schrödinger, Inc., 120 West 45th Street, New York, NY 10036 USA

**Keywords:** RNA, Drug development, Small molecules, Molecular biology

## Abstract

The MUSASHI (MSI) family of RNA binding proteins (MSI1 and MSI2) contribute to a wide spectrum of cancers including acute myeloid leukemia. We find that the small molecule Ro 08–2750 (Ro) binds directly and selectively to MSI2 and competes for its RNA binding in biochemical assays. Ro treatment in mouse and human myeloid leukemia cells results in an increase in differentiation and apoptosis, inhibition of known MSI-targets, and a shared global gene expression signature similar to shRNA depletion of MSI2. Ro demonstrates in vivo inhibition of c-MYC and reduces disease burden in a murine AML leukemia model. Thus, we identify a small molecule that targets MSI’s oncogenic activity. Our study provides a framework for targeting RNA binding proteins in cancer.

## Abstract (web summary)

The RNA binding protein MUSASHI-2 (MSI2) is a potential therapeutic target for acute myeloid leukemia. Here the authors identify a small molecule inhibitor of MSI2 and characterize its effects in a murine leukemia model.

## Introduction

RNA-binding proteins (RBPs) play critical roles in cell homeostasis by controlling gene expression post-transcriptionally. Ribonucleoprotein complexes are essential for all steps of mRNA processing including splicing, polyadenylation, localization, stability, export and translation^1^. The contribution of RBPs to tumorigenesis (e.g., SRSF2, SF3B1, MSI, and SYNCRIP), through genetic perturbation or epigenetic dysregulation, has been found in a variety of human cancers^2–8^. Deregulation of the MSI family of RBPs comprised of MSI1 and MSI2 was initially reported in gliomas^9^, medulloblastomas^10^ and hepatomas^11^. In addition, the MSI family has been implicated in aggressive forms of colorectal^12,13^, breast^14,15^, lung^16^, glioblastoma^17^, pancreatic^18,19^ and hematological malignancies. The *MSI2* gene was initially reported as a translocation partner with *HOXA9* in patients progressing from chronic myelogenous leukemia to blast crisis (CML-BC)^20^. More recently, other rare genetic alterations in leukemia patients involving *MSI2* included *EVI1, TTC40, and PAX5*^21–23^. *MSI2* is the dominant family member in the blood and is expressed in 70% of AML patients^24,25^. It correlates with a poor clinical prognosis in multiple hematological malignancies^25–28^. Thus, MSI2 has been proposed as a putative biomarker for diagnosis in leukemia^24,25^.

The relevance and requirement for MSI2’s function in leukemia was demonstrated by a series of depletion and overexpression studies in both mouse and human systems. Initial studies found that MSI2 was required for the initiation and maintenance of BCR-ABL (CML-BC)^27^ driven myeloid leukemia and forced expression drove a more aggressive form of CML in mice. Subsequent studies found a role for MSI2 in maintaining the MDS stem cell in a NUP98-HOXD13 mouse model and inducible forced expression of MSI2 drove a more aggressive form of MDS/AML that was dependent on sustained MSI2 induction^28^. In addition, *Msi2* was shown to be required for leukemic stem cells (LSC) in a retroviral transplantation MLL-AF9 mouse model of AML^7,29^. Depletion of MSI2 with shRNAs resulted in reduced colony formation and proliferation followed by differentiation in CML-BC and AML cell lines^26,27^. We and others have found that MSI2 mediates its function as an RNA binding protein controlling translation of its target RNAs^7,27,30,31^.

Based on the genetic studies, small molecule antagonists for MSI2 should be developed as they could be used as molecular probes or as potential therapeutics^32^. However, many RNA-binding proteins have been considered “undrugabble” due to their lack of well-defined binding pockets. One strategy to block MSI function would be to inhibit its RNA binding activity. The MSI family contains two highly conserved RNA-recognition motifs (RRMs) in the N-terminal region^33^. The first RRM1 is the determinant for RNA binding specificity whereas RRM2, mainly adds affinity^34^. MSI preferentially binds UAG-containing sequences in human^34^ and the minimal binding consensus described for RRM1 mouse MSI1 is r(GUAG)^35^. A previous study identified small molecules that interfered with MSI2 binding to RNA^36^. Here we describe the identification and characterization of one of the validated hits in our screen: Ro 08–2750 (Ro). Using biochemical and structural approaches, we find that Ro binds to the MSI2 RRM1 RNA-binding site, inhibits MSI RNA-binding activity and the regulation of downstream oncogenic targets. Furthermore, we demonstrate that Ro has efficacy in inhibiting myeloid leukemogenesis in both in vitro and in vivo models.

## Results

### Ro binds to MSI2 and inhibits its RNA-binding activity

**Fig. 1:**
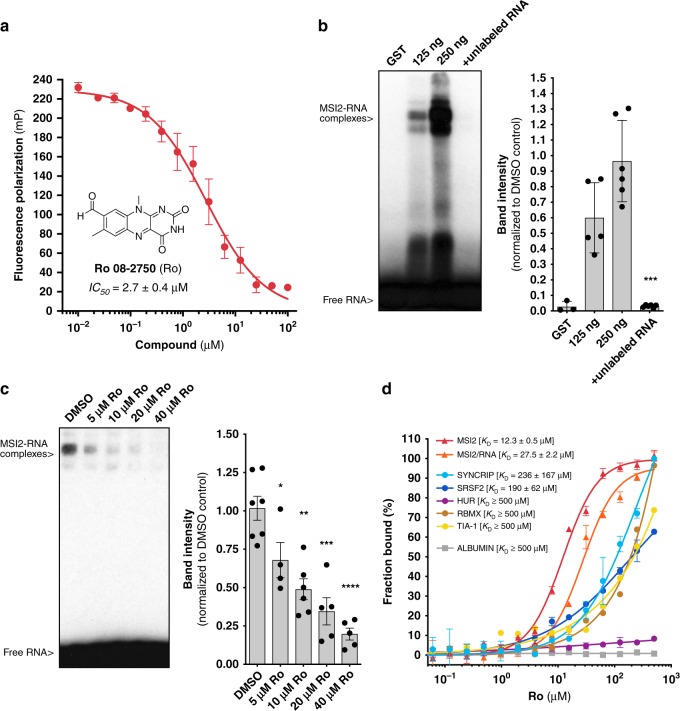
Ro 08–2750 (Ro) is a selective MSI RNA-binding activity inhibitor. **a** Fluorescence polarization secondary validation of Ro 08–2750 (Ro) *IC*_50_ for MSI-RNA binding inhibition in 384-well format. Seven independent experiments performed in duplicate ± standard error mean (s.e.m.) are shown; (**b**) Representative Electrophoresis Mobility Shift Assays (EMSA) for GST-MSI2 and GST-MSI2 proteins (125 and 250 ng) using biotinylated-RNA oligo in the absence or presence of unlabeled RNA (left); quantification of MSi2-RNA complexes of five independent experiments ± s.e.m. is shown in bar graph (right); (**c**) EMSA for GST-MSI2 (125 ng) in the presence of increasing concentrations of Ro (5 to 40 μM); quantification of RNA-protein complexes of at least four independent experiments ± s.e.m. is shown in bar graph (right); (**d**) Microscale Thermophoresis (MST) assay with interaction of Ro and GST-MSI2 (red), GST-MSI2/RNA complexes (orange) and the RRM-based RBP controls GST-SYNCRIP (light blue), GST-SRSF2 (dark blue), GST-HUR (*purple*), GST-RBMX (brown) and GST-TIA−1 (*yellow*) and non-RBP control bovine serum ALBUMIN (gray). Ro concentrations ranged from 0.0153 to 500 μM. Affinity (*K*_D_) values ± s.e.m. (μM) of at least three independent experiments are shown as percentage of fraction bound. For **b** and **c**: two-tailed Paired *t*-test; *p < 0.05; **p < 0.01, ***p < 0.005, ****p < 0.001. Source data are provided as a Source Data file

In order to identify a putative MSI RNA binding antagonist, we previously performed a fluorescence polarization (FP)-based screen using recombinant MSI1 and MSI2 and a consensus target RNA with a library of 6208 compounds^36^. We selected Ro 08–2750 (Ro) based on its RNA-binding inhibition of both MSI1 and MSI2^36^. MSI2 RNA-binding inhibition was confirmed by FP (*IC*_50_ of 2.7 ± 0.4 μM) (Fig. 1a). We then used a chemiluminescent Electrophoresis Mobility Shift Assay (EMSA) to quantify MSI2-RNA complexes in vitro. MSI2 binding could be competed with unlabeled RNA or by increasing concentrations of Ro (Fig. 1b, c). We then performed MicroScale Thermophoresis assay (MST) and found that Ro directly interacted with MSI2 with a *K*_D_ of 12.3 ± 0.5 μM (Fig. 1d). This interaction was then narrowed down to just the RNA-recognition motif 1 (RRM1), (Supplementary Fig. 1a). Also, the interaction with MSI2 could be competed with the addition of target RNA (*K*_D_ of 27.5 ± 2.6 μM). These data suggest that Ro directly interacts with the MSI2 RRM1 and competes with RNA binding.

To determine the selectivity for Ro binding to MSI2, we tested additional proteins and RNA. We first tested binding to a universal transport protein with hydrophobic binding pockets that is known to interact with small molecules non-specifically (bovine serum ALBUMIN) and found negligible binding (>500 μM), (Fig. 1d). We then tested several RBPs with evolutionarily conserved RRMs, such as SRSF2, HUR, RBMX, TIA-1 and SYNCRIP which was found to share many of MSI2’s mRNA targets (Supplementary Fig. 1b)^4^. Ro bound SRSF2 and SYNCRIP with a 15.5-fold and a 19.2-fold higher *K*_D_ than for MSI2 and the other RBPs had *K*_D_ ≥ 500 μM (Fig. 1d). Since isoalloxazine derivatives have been demonstrated to directly bind RNA^37^, we then tested Ro’s interaction to various RNAs. Using Isothermal Titration Calorimetry (ITC), we found no binding to RNAs that contained MSI2 motifs or to poly(A) RNA (Supplementary Fig. 1c–d), whereas palmatine interacted with similar affinity as previously reported^38^ (*K*_a_ = 4.17 ± 0.2 × 10^6^ M^−1^; *ΔH°* = −7.8 ± 0.1 × 10^3^ cal mol^−1^; *K*_D_ = 0.24 ± 0.01 μM). Ro’s affinity to MSI2 was also confirmed by ITC (*K*_a_ *=* 7.54 ± 0.15 × 10^4^ M^−1^*; ΔH°* = −1.95 ± 0.15 × 10^6^ cal mol^−1^*; K*_*D*_ *=* 13.3 ± 0.27 μM) (Supplementary Fig. 1e). Overall, these data suggest that Ro is selectively binding to MSI2.

### Ro interacts with the RNA recognition site of MSI2 RRM1

**Fig. 2:**
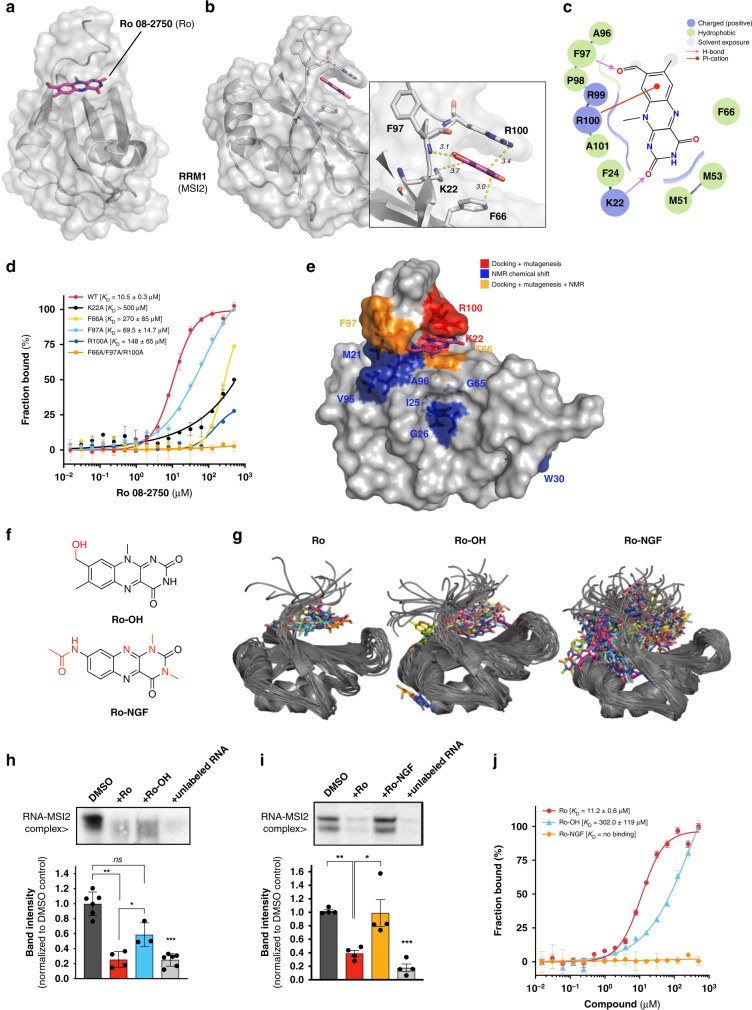
Ro interacts with the RNA-recognition motif 1 (RRM1) of MSI2. **a** Protein surface front view of the docked Ro in the RNA-binding site of human MSI2 RRM1 based on the X-ray diffraction crystal structure obtained at 1.7 Å resolution (RCSB PDB 6DBP); (**b**) Lateral and close up (*inset*) view of Ro with the relevant interaction residues (K22, F66, F97 and R100, full-length residue #) and distances (Å) between them and Ro’s closest atoms; (**c**) 2D representation of residues involved in Ro binding with K22 (H-bonding), F66 (hydrophobic stacking), F97 (H-bonding) and R100 (π-cation interaction) from RRM1; (**d**) Microscale Thermophoresis (MST) assay of Ro with full-length GST-MSI2 WT (red), K22A (black), F66A (yellow), F97A (cyan), R100A (navy blue) and Triple (F66A/F97A/R100, orange). *K*_D_ values ± standard deviation (s.d.) (μM) of at least three independent experiments (as percentage of fraction bound); (**e**) Protein surface representation with RRM1 residues interacting with Ro identified by docking and mutagenesis analysis (red) and residues with indicated chemical shift changes by NMR after Ro interaction (blue). In light orange, residues identified by both experimental approaches. **f** Chemical structures of Ro analogs used in **g**–**j** panels. Ro-NGF (Neural Growth Factor-NGF-inhibitor, *K*_D (NGF)_ = 1.7 × 10^–6^ M) and Ro-OH; (**g**) The cluster centers for Ro (left), RoOH, (center) and Ro-NGF (right), derived using regular spatial clustering with a ligand RMSD cutoff of 1 Å. Ro-NGF (right) indicating a larger number of clusters than Ro (left) or RoOH (center). **h** Representative EMSA for GST-MSI2 (125 ng) in the absence (DMSO) or presence of Ro (20 μM), Ro-OH (20 μM) or unlabeled RNA oligo (1 μM) and quantification of RNA-protein complexes (mean ± s.e.m. of three independent experiments); (**i**) Representative EMSA for GST-MSI2 (125 ng) in the absence (DMSO) or presence of Ro (20 μM), Ro-NGF (20 μM) or unlabeled RNA oligo (1 μM) and quantification of RNA-protein complexes (bar graph: see **h**); (**j**) MST assays with interaction of Ro, Ro-OH and Ro-NGF and GST-MSI2 WT. *K*_D_ values ±  s.d. (μM) of at least three experiments are shown as percentage of fraction bound; For **g** and **h**, two-tailed Paired *t*-test; *ns*, not significant, *p < 0.05, **p < 0.01, ***p < 0.005. Source data are provided in a Source Data file

To study how Ro interacts with MSI2, we obtained a crystal structure of the *apo* human MSI2 RRM1 at 1.7 Å resolution (Table 1, RCSB PDB accession code 6DBP) after unsuccessful co-crystallization attempts. We performed docking analysis to identify a putative binding region (Fig. 2a, b and Supplementary Fig. 2a, b). Based on Ro’s ability to compete for MSI-RNA complexes, we hypothesized that the binding site is likely to be shared with the RNA binding site. Thus, we predicted a stacking interaction with F66 and R100 with Ro (Fig. 2b). Also, the K22 side chain and the NH backbone group from F97 formed stabilizing H-bonds with the oxygens from the aldehyde in the C_2_ position in the pyrimidine ring and the aldehyde from the opposite ring (Fig. 2b, c). A 2D representation indicates that the R100 forms a non-covalent π-cation and two H-bonding interactions with K22 and F97 (Fig. 2c).

**Table 1:** Data collection and refinement statistics (molecular replacement)

	Crystal 1
Data collection	
Space group	P2_1_
Cell dimensions	
* a*, *b*, *c* (Å)	30.32, 64.41, 38.39
α, β, γ (°)	90.00, 108.9, 90.00
Resolution (Å)	30–1.6 (1.66–1.60)
* R*_pim_	0.028 (0.163)
* I* / σ*I*	21.2 (3.2)
Completeness (%)	91.5 (60.6)
Redundancy	2.7 (1.8)
Refinement	
Resolution (Å)	30–1.6
No. reflections	16762
* R*_work_ / *R*_free_	0.173/ 0.211
No. atoms	1468
Protein	1328
Ligand/ion	0
Water	140
* B*-factors	20.0
Protein	19.5
Ligand/ion	N/A
Water	29.6
R.m.s. deviations	
Bond lengths (Å)	0.007
Bond angles (°)	0.863

Diffraction data collected from a single crystal. Values in parentheses are for highest-resolution shell

To directly test our docking model, we mutated the putative interacting residues (K22A, F66A, F97A and R100A) and determined their ability to bind to Ro. This resulted in significantly reduced binding (F97A *K*_D_ 69.5 ± 14.7 μM, R100A *K*_D_ 148 ± 65 μM, F66A and K22A *K*_D_ > 200 μM compared with 10.5 ± 0.3 μM for WT) (Fig. 2d). The triple mutant (F66A/F97A/R100A) was incapable of binding to Ro. Importantly, the single mutations did not disrupt RNA binding to MSI2 whereas the triple mutant completely inhibited its activity (*K*_D_ > 50 μM) (Supplementary Fig. 2c). These data support our docking since the single mutants demonstrated reduced Ro binding activity without altering RNA binding.

To provide additional evidence for the docking model, we performed NMR chemical shift analysis of ^15^N-labeled RRM1 and observed three main regions with structural changes by titrating MSI2 with Ro: (1) K22 region (with M21, I25, G26 and W30 shifts), (2) F66 region (with S61, G65, F66, and V67 shifts) and (3) F97 region (showing V95, A96, F97, and R100 shifts) (Fig. 2e and Supplementary Fig. 2d, e). Thus, our mutagenesis and NMR chemical shift analysis support our docking model.

To test structure–activity relationships, we evaluated two Ro related molecules (Ro-NGF and Ro-OH, Fig. 2f). The first analog, Ro-NGF, previously synthesized and described by Eibl et al.^39^, was selected to determine if Ro’s activity was related to its anti-Neural Growth Factor (NGF) activity, as this compound showed the highest affinity for neural growth factor (NGF) (*K*_D [NGF]_ = 1.7 μM) in its compound series (Supplementary Data 1). The second analog, Ro-OH, was synthesized by reduction of the Ro aldehyde to the corresponding alcohol, providing a single alteration to the structure (Supplementary Fig. 3a–c). Initial structural analysis of our docked model suggested that the Ro-OH–MSI complex lacks the R100 π-cation interaction and that Ro-NGF binds MSI in a position displaced from the RNA-binding core (Supplementary Fig. 4a–d) as compared with Ro. We computed similar binding free energies (*ΔG*_bind_) for the three ligands (Ro, Ro-OH and Ro-NGF, −5.5 and −6.1 versus −5.1 kcal mol^−1^ for Ro-NGF) (Supplementary Fig. 4e). Both MSI2 and ligands adopted a heterogeneous ensemble of conformations and binding poses, with the protein-ligand complex predicted to undergo a slight conformational change upon binding of Ro and Ro-OH (Supplementary Fig. 4f). Free energy calculations for all three small-molecules suggest that the Ro-NGF–MSI complex adopts a much more diverse set of conformations (as measured by conformational clustering of the fully interacting alchemical state) than the complexes with Ro-OH or Ro (Fig. 2g). The Ro–MSI complex demonstrated the fewest clusters, with the top three clusters accounting for 92.7% of the sampled configurations (Supplementary Fig. 4g). The Ro-OH–MSI complex showed a larger number of clusters, with the four clusters accounting for 49.1% of sampled configurations, indicating a greater degree of heterogeneity than Ro (Supplementary Fig. 4h).

To experimentally validate these predictions, we performed EMSA of GST-MSI2 competing RNA with Ro-OH or Ro-NGF, comparing their potency with Ro and unlabeled RNA as positive controls. Accordingly, Ro-OH had reduced activity compared with Ro (∼30–40% versus 65–75%, *p* < 0.05), whereas Ro-NGF was completely unable to disrupt MSI2-RNA complexes (Fig. 2h, i). These results were confirmed by a FP assay with Ro-OH inhibiting with 12.5-fold less potency than Ro, and Ro-NGF failing to inhibit RNA-binding activity (Supplementary Fig. 4i). Furthermore, in MST assays, Ro-OH showed a 27-fold lower affinity than Ro (*K*_D_ of 302.0 ± 119 μM for Ro-OH versus 11.2 ± 0.6 μM for Ro) for GST-MSI2, whereas Ro-NGF failed to demonstrate any interaction up to 500 μM (Fig. 2j). Thus, our structural and biochemical experimental data support the conclusion that Ro and MSI2 interact via the RRM/ RNA binding site and that the drug can displace RNA from its binding site, thus likely inhibiting MSI-related RNA regulation.

### Ro demonstrates activity in murine MLL-AF9 leukemic cells

**Fig. 3:**
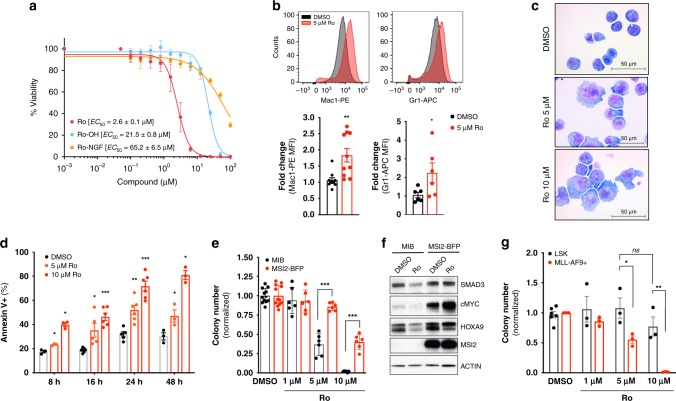
Ro 08–2750 increases differentiation and apoptosis in myeloid leukemia cells. **a** Cytotoxicity assay (Cell-Titer Glo) of Ro (*red*), Ro-OH (*cyan*) and Ro-NGF (*orange*) in MLL-AF9+ BM cells. 50% Effective Concentration (*EC*_50_) values, average of at least three independent experiments ± standard deviation are shown. **b** Flow cytometry representative histograms of DMSO (*gray*) and 5 μM Ro (*red*) treated MLL-AF9+ BM cells with myeloid differentiation markers (Mac1 and Gr1); bar graphs (*below*) show average (fold change increase) ± standard error mean of three independent experiments, performed in triplicate. Paired *t*-test, *p < 0.05; **p < 0.01. **c** Representative immunocytochemistry images of cytospun MLL-AF9 + BM cells control (DMSO) or Ro treated (5 and 10 μM) and stained by Eosin Y and Methylene Blue/ Azure A. Scale, 50 μm. **d** Apoptosis analysis by Annexin V+ (% population) for MLL-AF9 + BM cells cultured in absence (DMSO, *black*) or presence of Ro 5 μM (*light red*) or 10 μM (*red*). Results represent at least three independent experiments ± s.e.m. **e** Colony Formation Unit (CFU) assay of MLL-AF9 + Puro-CreER + Msi1^*fl/fl*^ Msi2^*fl/fl*^ BM cells transduced with MSCV-IRES-BFP (MIB, control) or MSCV-IRES-MSI2-BFP (MSI2-BFP) retroviral vectors after Ro treatment 1, 5 and 10 μM. Results represent the average ± s.e.m. of colony numbers of at least five experiments performed in duplicate. **f** Representative immunoblot of MLL-AF9 + Puro-CreER + Msi1^*fl/fl*^ Msi2^*fl/fl*^ BM MIB (*black bars*) and MSI2-BFP (*red bars*) cells (used in panel **e**) after DMSO or 10 μM Ro treatment for 4 h. β-ACTIN, loading control. **g** CFU assay of Lin^-^Sca^+^cKit^+^ (LSK) versus MLL-AF9+ BM cells demonstrates Ro 08–2750 therapeutic window. Results represent the average ± s.e.m. of colony numbers of three experiments performed in duplicate. Two tailed Paired *t*-test (**b**, **d**, **e** and **g**), *p < 0.05, **p < 0.01, ***p < 0.005. Source data are provided as a Source Data file

To test the cellular effect of Ro in a murine AML leukemia model, we used previously established MLL-AF9 expressing leukemic bone marrow (BM) cells from secondary transplants^7^. Consistent with an on-target effect on MSI inhibition and in agreement with the RNA-binding activity inhibition assays, Ro effectively inhibited leukemia cell proliferation (half-effective concentration, *EC*_50_ = 2.6 ± 0.1 μM). By comparison, the analogs that failed to interact with MSI2 had a diminished effect (Ro-OH *EC*_50_ = 21.5 ± 0.8 μM; Ro-NGF > 50 μM), suggesting that the antiproliferative effect is due to the ability of Ro to inhibit MSI2 RNA binding-activity (Fig. 3a). Treatment of cells with Ro resulted in an increase in differentiation at 5 μM dose and 48 h treatment as seen both quantitatively by flow cytometry (Fig. 3b) and by morphological analysis (Fig. 3c). We found a significant increase in apoptosis (Annexin V+ population as early as 8 h both at 5 and 10 μM) with the highest increase at 48 h and 10 μM Ro (Fig. 3d and Supplementary Fig. 5a). We then assessed how MSI2 overexpression affected the plating capacity of MLL-AF9+ BM cells in the absence or presence of Ro. MSI2 overexpressing cells formed 50% more colonies than control cells transduced with an empty vector. Treatment of cells with Ro resulted in reduced colony formation in control cells by >50% and ∼75% at 1 μM and 5 μM concentrations, respectively. Furthermore, MSI2-overexpressing leukemia cells demonstrated reduced activity at these doses (Fig. 3e). Consistently, MSI2’s translational direct targets^7,30^ SMAD3, c-MYC, and HOXA9 were reduced, whereas their abundance remained unaffected in cells that overexpressed MSI2 (Fig. 3f). Moreover, colony-forming ability was further rescued by overexpressing MSI2-Ro-binding mutants (K22A, F66A, F97A, and R100) (Supplementary Fig. 5b, c). Overall, these data further support that MSI2’s cell toxicity was related to MSI2 and its RNA binding activity. We also found that Ro blocked MLL-AF9+ BM colony formation at concentrations that did not affect the plating efficiency of normal Lin-Sca+ cKit+ (LSK) cells, indicating a potential therapeutic window between normal and malignant cells (Fig. 3g).

### Ro inhibits survival of human AML lines and patient cells

**Fig. 4:**
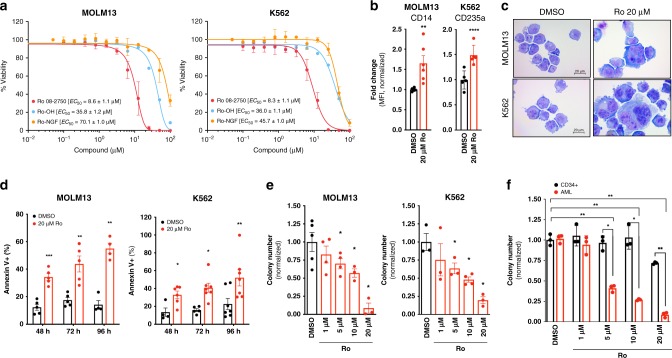
Ro 08–2750 treatment inhibits survival of human AML cell lines and patient cells. **a** Cytotoxicity assay (Cell-Titer Glo) of Ro, Ro-OH and Ro-NGF in MOLM13 and K562 cells. *EC*_50_ values average of three independent experiments ± s.d. is shown. **b** Mean Fluorescence Intensity (MFI) fold changes of CD14 (myeloid marker, MOLM13) and GlycA (Glycophorin-A, CD235a; erythroid marker, K562) after 48 h treatment with DMSO (control, black bars), Ro (red bars) at 20 μM. Data is normalized to DMSO control cells. Representative histograms for each marker are shown in Supplementary Fig. 6a. **c** Representative immunocytochemistry images of cytospun MOLM13 and K562 cells treated for 48 h with DMSO (control), Ro 20 μM and stained with Eosin Y and Methylene Blue/ Azure A. Scale, 20 μm. **d** Apoptosis analysis by Annexin V+ (% population). MOLM13 and K562 were cultured in DMSO (*black bars*) or in the presence of Ro 20 μM (red bars) for the indicated times and Annexin V positivity and 7AAD was measured. Results represent three independent experiments ± s.d. **e** CFU assay of MOLM13 and K562 in the presence of Ro at different concentrations (1, 5, 10, and 20 μM). Data is shown as average colony numbers (normalized to DMSO control) ± s.e.m. of at least three independent experiments. **f** CFU assay of cord-blood derived CD34 + HSPCs and AML patient BM cells. Data is shown as average colony numbers (normalized to DMSO) ± s.e.m. of three different blood donors for CD34 + and three independent AML patients. Two tailed Paired *t*-test (DMSO versus Ro treated, unless indicated with lines); *p < 0.05; **p < 0.01; ***p < 0.005, ****p < 0.001. Source data are provided as a Source Data file

To determine if Ro has activity against human myeloid leukemia, we first tested cytotoxicity effects in MOLM13 (AML, MLL-AF9+) and K562 (CML-BC, BCR-ABL+) cell lines^4,27^. Similar to the mouse leukemia cells, Ro demonstrated an anti-proliferative effect (*EC*_50_ ∼8 μM), whereas the two analogs (Ro-OH and Ro-NGF) revealed >4-fold weaker potency. Ro affected viability of CD34+ cord blood cells at an *EC*_50_ of ∼22 μM, 2.6-fold higher concentration than the human leukemia cell lines (Fig. 4a and Supplementary Fig. 6a). Ro induced myeloid differentiation and promoted apoptosis in both K562 and MOLM13 cells based on flow cytometry and morphology (Fig. 4b–d and Supplementary Fig. 6b–d) without any effect on differentiation on normal CD34+ cord blood cells (Supplementary Fig. 6e, f). Plating activity was >80% inhibited at the 20 μM Ro dose in the human AML cell lines (Fig. 4e). In addition, Ro demonstrated differential sensitivity in three AML patient samples in colony plating assays compared with normal human CD34+ cord blood cells (>50% inhibition in colony numbers at 5 μM compared with only a modest reduction at 20 μM Ro (Fig. 4f and Supplementary Data 2). These results suggest that Ro can induce toxicity in human myeloid leukemia cells with a (2-fold) level of selectivity compared with normal cells.

### Ro inhibits MSI2 RNA-binding and alters MSI2 gene signature

**Fig. 5:**
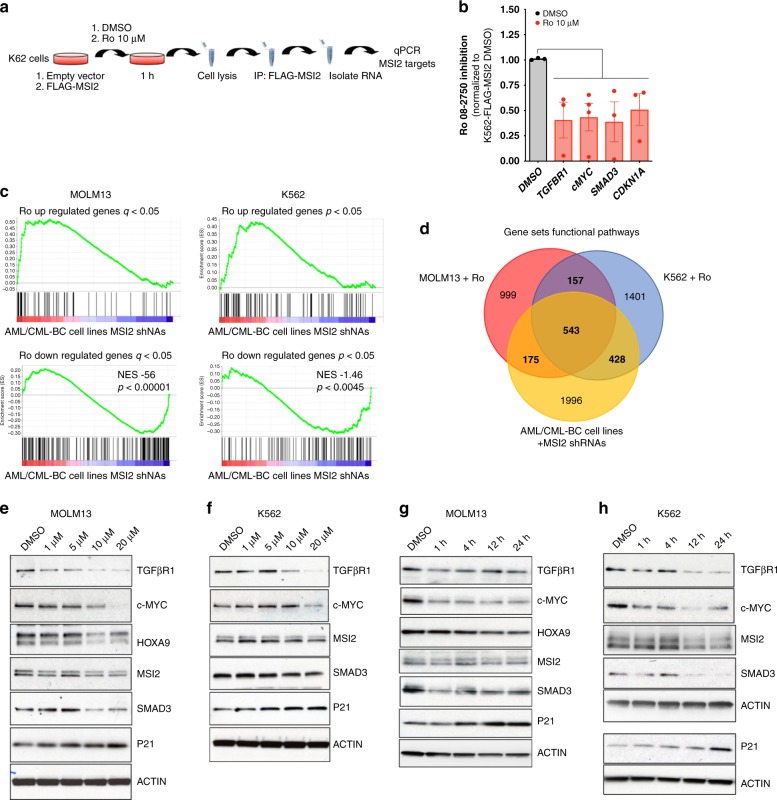
Ro 08–2750 treatment corresponds with MSI2 shRNA depleted gene signature. **a** Scheme of RNA-immunoprecipitation (IP) protocol followed with K562-MIG (MSCV-IRES-GFP) or FLAG-MSI2 overexpressing cells. **b** Ro 08–2750 inhibitory effect in the RNA-IP enrichment of MSI2 mRNA targets in K562-FLAG-MSI2 versus K562-MIG after 1 h treatment at 10 μM. Data is shown as average of inhibition effect (normalized to DMSO cells) ± s.e.m. of four independent experiment. **c** Upregulated and down-regulated gene sets obtained by RNA-seq analysis after 20 μM Ro 4 h treatment in MOLM13 and K562 cells showing identical signature as previously obtained using shRNA against MSI2 in CML-BC and AML lines^27^. **d** Venn diagram showing gene Set Enrichment Analysis (GSEA) overlap between MOLM13 (*red*), K562 (blue) (after 20 μM Ro 4 h treatment) and AML/CML-BC cell lines MSI2 depleted with shRNAs (*yellow*) from Kharas et al.^27^. Bold values inside brackets below each grup are total gene sets numbers. **e** Representative immunoblot for MOLM13 treated with Ro at different concentrations (1, 5, 10, and 20 μM) for 4 h showing expression of MSI2 targets. HOXA9 is not expressed in this BCR-ABL+ (CML-BC) leukemia cell line. **f** Representative immunoblot for K562 treated with Ro at different concentrations for 4 h showing expression of MSI2 targets. **g** Representative immunoblot for MOLM13 treated with Ro 20 μM at different time points (1, 4, 12 and 24 h) showing expression of same MSI2 targets as in panel **e**. **h** Representative immunoblot for K562 treated with Ro 20 μM at different time points with an effect on MSI2 targets. P21 and β-ACTIN from a different representative gel are shown. Source data are provided as a Source Data file

To further investigate the effect and mechanism of action of Ro, we initially performed RNA immunoprecipitation (RNA-IP with FLAG) experiments on K562-MIG (empty vector) and K562-FLAG-MSI2 (MSI2 overexpressing) cells (Fig. 5a). By incubating the drug at 10 μM (∼*EC*_50_) for 1 h with the cells, we could detect a significant decrease in MSI2 mRNA binding targets (*TGFBR1*, *c-MYC*, *SMAD3*, *CDKN1A*) (Fig. 5b). These data suggest that Ro can block MSI2 binding to target mRNAs in a cellular context at a short time-point.

To globally assess the proximal effect of Ro treatment on the transcriptional program, we then performed RNA-sequencing on MOLM13 and K562 cells after 4 h of treatment. Ro incubation resulted in modest but significant gene expression changes in both the MOLM13 and K562 AML cells (59 upregulated, 221 downregulated and 111 upregulated, 164 downregulated, respectively; FDR < 0.05) (Supplementary Data 3–4). Most importantly, this Ro signature enriched for the gene expression profiling after shRNA mediated depletion of MSI2 in CML-BC (AR-230 and LAMA84) and AML cell lines (THP1 and NOMO-1) (Fig. 5c and Supplementary Data 5)^27^. To annotate the functional pathway overlap with Ro treatment in both cell lines and MSI2 shRNA depletion, we performed gene-set enrichment analysis (GSEA)^40^ on all 4,733 curated gene sets in the Molecular Signatures Database (MSigDB, http://www.broadinstitute.org/msigdb) combined with 92 additional relevant gene sets from our experimentally derived or published hematopoietic self-renewal and differentiation signatures^30,40^. Interestingly, we observed an overlap of MSI-associated signatures from our previous dataset and enrichment with MSI1 direct mRNA targets from the intestine (Supplementary Fig. 7a and Supplementary Data 6–11)^4^. Moreover, we observed a ~70% overlap of the functional pathways between each individual cell line and the pathways altered after shRNA depletion of MSI2 (Fig. 5d). Among these shared pathways, 76% (543 out of 717) overlapped in MOLM13 compared with K562 cells treated with Ro, which included c-MYC, mRNA-related, and leukemia-associated gene sets (Fig. 5d and Supplementary Data 12). Thus, Ro treatment after a short administration recapitulated a large portion of the MSI2-associated gene expression program.

To determine how Ro affects previously determined MSI targets, we treated both K562 and MOLM13 cells with increasing concentrations of Ro (up to 20 μM at 4 h). In previous studies, MSI was demonstrated to maintain the protein levels of TGFβR1, c-MYC, SMAD3, and HOXA9^7,30^ while suppressing P21 abundance^41^. Consistent with this, we observed a significant dose dependent reduction of TGFβR1, c-MYC, SMAD3, HOXA9 and an increase P21, while the non-target control β-ACTIN remained unchanged (Fig. 5d, e). In addition, Ro could inhibit MSI2 targets in a time-dependent manner with c-MYC, a short half-life protein, being reduced in 1 h of treatment (Fig. 5f, g). In support of Ro altering translation of specific MSI2 targets but not generally inhibiting global translation, we found equivalent global protein synthesis after drug treatment as assessed by *O*-propargyl-puromycin incorporation (Supplementary Fig. 7b). As previously noted by RNA-sequencing, there were modest effects on the mRNA expression of MSI2 targets by qPCR (Supplementary Fig. 7c) suggesting that Ro mainly influences its direct targets through a post-transcriptional mechanism. Thus, these results support our hypothesis that Ro acts in the MSI-related translational program.

### Ro inhibits leukemogenesis in a myeloid leukemia model in vivo

**Fig. 6:**
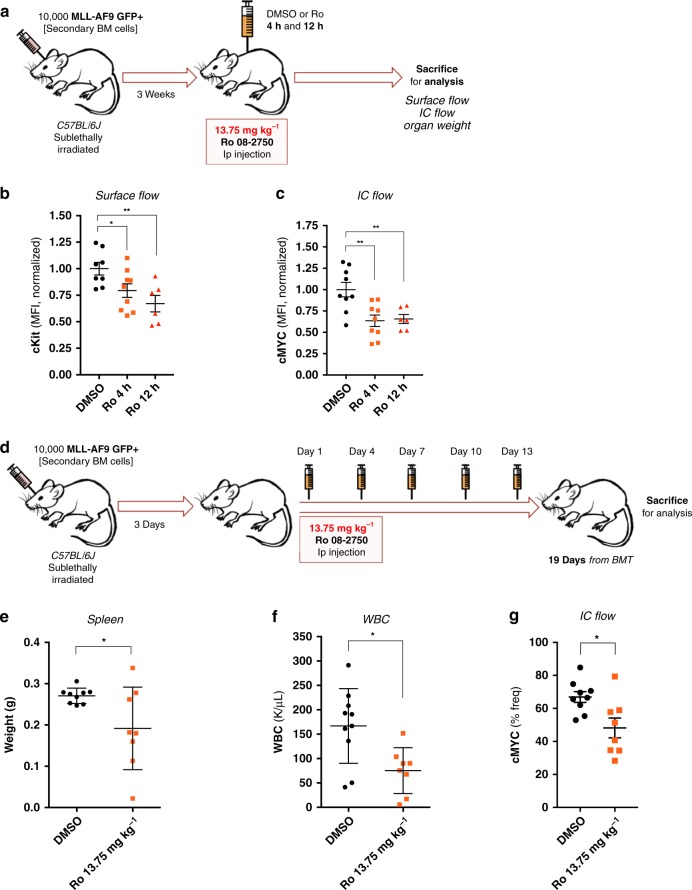
Ro 08–2750 demonstrates efficacy in an MLL-AF9 in vivo model. **a** Scheme of pharmacodynamics marker experiments with Ro short-time points performed with MLL-AF9 + secondary BM cells. 10,000 MLL-AF9 GFP + cells were transplanted and, after 3 weeks, mice were injected with DMSO or Ro (13.75 mg kg^−1^) and were sacrificed for analysis after 4 h and 12 h. **b** Surface flow analysis of c-Kit receptor in spleen cells of Ro at 4 h and 12 h *versus* DMSO treated mice. Results are represented as MFI of cKit-PE-Cy7 normalized to DMSO group. Each data point is an independent treated mouse. Mean ± s.e.m. is shown. **c** Intracellular (IC) flow analysis of c-MYC expression in spleen cells of Ro at 4 h and 12 h *versus* DMSO treated mice. Results are represented as MFI of c-MYC normalized to DMSO group. Each data point is an independent treated mouse. Mean ± s.e.m. is shown. **d** Scheme of in vivo Ro treatment in MLL-AF9 + model of myeloid leukemia. 10,000 MLL-AF9 GFP + cells were transplanted and after 3 days, mice were injected with DMSO or Ro 13.75 mg kg^−1^ (in DMSO) intraperitoneally (IP) at days 1, 4, 7, 10, and 13 (one day *on*, two days *off* drug). At day 19 of treatment, mice were sacrificed for organ weight and flow cytometry analysis of disease burden and MSI2 target, c-MYC. **e** Spleen weights at time of sacrifice. Results are represented in weight (g) and each data point represents an individual DMSO or Ro treated mouse. **f** White blood cell (WBC) counts (K μL^−1^) at time of sacrifice. Each data point represents an individually treated mouse. **g** Intracellular (IC) flow analysis of c-MYC expression in spleen cells of Ro versus DMSO treated mice. Results are represented as % frequency (% freq) of c-MYC + cells. Each data point is an independent treated mouse. Mean ± s.e.m. is shown. For all graphs, unpaired *t*-test; *p < 0.05, **p < 0.005. Source data are provided as a Source Data file

Finally, we sought to determine if Ro has in vivo efficacy using an aggressive murine MLL-AF9 murine leukemia model. Mice were treated with Ro at 13.75 mg kg^−1^ ip, the highest dose achievable due to limited compound solubility and the use of DMSO as an excipient. Acute treatment of Ro (4 and 12 h) reduced c-KIT protein abundance and intracellular c-MYC (Fig. 6a–c). To determine if Ro treatment could effect disease burden, we next treated a second cohort of animals and monitored them for disease progression for 19 days after transplantation (Fig. 6d). Ro administration every 3 days was well tolerated (Supplementary Fig. 8a) with mice exhibiting little to no weight loss and equivalent red blood cell, platelet, mean corpuscular volume, hematocrit, and hemoglobin counts compared with the non-treated group (Supplementary Fig. 8b–f). Using healthy mice, we also reported no changes in liver enzymes 24 h after Ro treatment (Supplementary Fig. 8g). Although there was no change in leukemia latency in this very aggressive model, disease progression was assessed in both treated and control groups when control mice and treated mice succumbed to disease (day 19 post-transplantation). The treated group exhibited a significant reduction in spleen weights (Fig. 6e), white blood cell counts (Fig. 6f) and c-MYC levels compared with the control group (Fig. 6g). These data support the concept that targeting MSI in vivo could have therapeutic efficacy in AML.

## Discussion

Inhibiting MSI RNA-binding activity could represent a novel therapeutic avenue in both hematological malignancies and solid cancers. Our previous FP-based screen identified compounds that inhibit MSI binding to RNA^36^. Here, we characterize Ro 08–2750 (Ro) as a selective MSI inhibitor with biochemical, structural, and cellular validation linking the compound to the inhibition of the MSI program. Ro falls in the low micromolar range of activity, in line with other RBP associated inhibitors^42–44^. We validated Ro as a MSI2 RNA-binding inhibitor with biophysical and biochemical assays. We obtained a high-resolution crystal structure of the MSI2 RRM1 which allowed us to utilize a newly developed computational molecular modeling algorithm and perform docking analysis. This docking analysis was supported through the identification of key interacting residues within the known RNA binding region finding that was confirmed by NMR chemical shift analysis. Both our novel crystal structure and the computational tools will be useful for the discovery and development of small-molecule RBPs inhibitors. We found that a single chemical reduction of Ro drastically decreased its activity in both in biochemical and in vitro cell based assays. Utilizing a related compound with high affinity binding to NGF, we found that it no longer bound MSI2 and poorly inhibited leukemia cell growth. Further studies involving medicinal chemistry with heterocycle isoalloxazines or pteridine-derived compounds could help identify more selective and potent MSI-inhibitors. Despite the potential of this class of molecules to interact with structured RNA motifs^37^, no direct RNA binding to MSI RNA probes or poly(A) was found for Ro.

Other groups have identified agents that have putative MSI1 inhibitory activity. The natural phenol extracted from cottonseed ((−)-gossypol) was shown to reduce MSI1 to bind RNA^43^ but this interaction was not tested for selectivity. (−)-Gossypol has been considered to be a pan-active compound that has hit in multiple HTS screens^45,46^ and assigned to have activity against Bcl-2^47^. MSI1 activity was also inhibited by ω−9 monounsaturated fatty acids (e.g. oleic acid), allosterically binding and inducing a conformational change that prevents RNA to bind^48^. It remains unclear if (−)-gossypol or oleic acid have a broader RNA binding protein inhibitor activity as they were not directly tested against other RBPs^43,48^. We found that Ro could demonstrate differential binding activity to MSI2 compared to five different RRM-based RBPs, Ro’s effect on colony formation and direct targets could be rescued by MSI2 overexpression and by mutants of MSI2 that bind poorly to the inhibitor. Moreover, we observed a strong enrichment for the MSI2 shRNAs gene expression signature, associated functional pathways, inhibition of MSI2 binding of target mRNAs and reduced abundance of MSI2 direct targets after Ro treatment. In contrast to other general translational inhibitors^49^, Ro did not alter global translation. These data suggest that Ro could be used to probe the acute effects of MSI inhibition in a variety of cellular contexts and cancer models.

It is also important to note that Ro inhibits both MSI1 and MSI2 and although MSI1 is expressed at low levels in myeloid leukemia it could still be blocking residual MSI1 activity. Moreover, in other models such as the intestine where both factors act redundantly^12^, dual inhibition could provide a powerful therapeutic strategy. Based on the close conservation of the RRMs between the two proteins it might be challenging to design MSI1 or MSI2 selective inhibitors.

We demonstrated a therapeutic index for Ro in human AML patient samples versus cord-blood derived CD34+ human stem and progenitor cells. Despite the challenges for in vivo administration, we reduced the disease burden in an aggressive MLL-AF9 leukemia model and decreased c-MYC levels without overt toxicity. Interestingly, it has previously been shown that MSI2 can contribute to chemotherapeutic resistance in different cancer models^50,51^. Future studies could examine if combination therapies could provide additional efficacy.

This study identifies and characterizes Ro 08–2750 as a compound selectively inhibiting the oncogenic RNA-binding activity of MSI in myeloid leukemia. It will be important to use this compound (or other chemical derivatives) to test their efficacy in other cancer models and on MSI function related to normal physiology. We suggest that Ro provides the rationale for developing more potent compounds with improved clinical utility for the treatment of cancers that are dependent on the MSI family. In addition, as there are hundreds of RRM containing RNA binding proteins, targeting an RRM motif to block RNA activity with Ro represents a valuable proof of concept for the general inhibition of this class of RNA regulators. Thus, we provide a framework to identify and test novel RNA binding protein inhibitors in cancer.

## Methods

### Purification and culture of cord blood derived HSPC-CD34+ cells

Mononuclear cells were isolated from cord blood using Hetarstach solution (6% Hetastarch in 0.9% NaCl) and Ficoll-Hypaque Plus density centrifugation. CD34+ Hematopoietic Stem and Progenitor Cells (HSPCs) were subsequently purified by positive selection using the Auto MACS Pro Separator and isolation kit (Miltenyi) and were cultured in Iscove’s modified Dulbecco’s medium (IMDM, Cellgro), 20% BIT 9500 medium (Stem Cell Technologies) supplemented with SCF (100 ng ml^−1^), FLT-3 ligand (10 ng ml^−1^), IL-6 (20 ng ml^−1^), and TPO (100 ng ml^−1^) as the basic culture. All cytokines were purchased from Peprotech, NJ.

### Viral transduction of murine MLL-AF9 leukemia and normal cells

Tibia, femurs, pelvis, and arm bones from leukemia or C57BL/6 wild type mice (10–12-weeks-old) were harvested, crushed, filtered, and subjected to red blood cell lysis (Qiagen). To isolate c-Kit^+^ cells, bone marrow cells were incubated with anti-CD117 microbeads (Miltenyi Biotec), according to manufacturer’s instructions, and then subjected to positive selection using autoMACS Pro Separator. For MLL-AF9+ BM cells, thawed vials from previously established secondary transplants^7^ were used. All murine cells were cultured and transduced in RPMI with 10% FBS and cytokines SCF (10 ng ml^−1^), IL-3 (10 ng ml^−1^), and IL-6 (10 ng ml^−1^) and GM-CSF (10 ng ml^−1^). For MSI2 WT or single mutants (K22A, F66A, F97A, R100A) overexpression, cells were spinfected with viral supernatant containing MSCV-IRES-BFP, MSI2-IRES-BFP, or mutants construct’s (see Cloning section).

### Colony forming unit assays

A total of 10,000 leukemic MLL-AF9 BM cells or c-Kit enriched normal stem cells (Lin-Sca+Kit+) were plated on methylcellulose-based culture media (MethoCult^TM^ GF M3434 Stem Cell Technologies). Colonies were scored every five days for leukemia cells and every seven to nine days for normal c-kit-enriched bone marrow cells. For human cells, 5000 of the leukemia cel lines K562 (CML-BC) or MOLM13 (AML) and 10,000 of HSPCs CD34+ or AML patient cells were obtained through Memorial Sloan Kettering Tumor Bank (IRB #18–272) plated (in duplicate) in methylcellulose (MethoCult^TM^ H4434 Classic, Stem Cell Technologies). CFU colonies in HSPCs CD34+ were scored 14 days after seeding. AML patient cells characteristics are shown in Supplementary Data 2. K562 (ATCC^®^ CCL-243) and MOLM13 (#ACC 554) lines were purchased from American Type Culture Collection (ATCC) and Deutsche Sammlung von Mikroorganismen und Zellkulturen (DSMZ), respectively, authenticated by Genetica, and tested negative for mycoplasma contamination.

### Flow cytometry analysis

To monitor the differentiation status, 200 K MLL-AF9 BM cells DMSO or Ro treated (during 24 and 48 h) were stained with the following antibodies: anti-CD11b (Mac1)-PE (clone M1/70, #101208, BioLegend), anti-Ly-6G (Gr1)-APC (clone RB6–8C5, #17–5931–82, eBioscience), and anti-CD117 (c-Kit)-APC-Cy7 (clone 2B8, #105826, BioLegend). For the human cell lines differentiation, we used two panels: (1) anti-CD14-PE (clone M5E2, #555398, BD Pharmingen), anti-CD13-APC (clone TUK1, #MHCD1305, Life Technologies); (2) anti-CD71-FITC (clone CY1G4, #334104, BioLegend), anti-CD235a (Glycophorin A)-PE (clone YTH89.1, #MA5–17700, Invitrogen). For CD34+ differentiation, we used anti-CD13-FITC (clone TUK1, #MHCD1301, Life Technologies) and anti-CD14-PE (same as above). All samples were stained for 20 min in the dark, washed once with PBS 1× and re-suspended in RPMI+ 2% FBS for analysis. For intracellular flow cytometry detection of c-MYC, 1–2 × 10^6^ cells were fixed in 2% paraformaldehide for 15 min, washed 2 times with 1× PBS and permeabilized with cold methanol and kept at −20 °C until use. For the staining, cells were washed twice in 1× PBS and stained in 100 μL final volume. c-MYC (5605, Cell Signaling Technology non-labeled primary antibody was incubated at 1/200 dilution for 1 h and labeled donkey anti-rabbit Alexa Fluor 568 (#A10042, Invitrogen) or goat anti-rabbit Alexa Fluor 647 (#A21245, Invitrogen) were used at 1/400 for 20–30 min. Cells were washed once with PBS 1× and re-suspended in RPMI+ 2% FBS for analysis. All flow cytometry analysis was performed in a LSRII or LSR Fortessa (BD Biosciences) and data was graphed by using FlowJo^TM^ version 10.4.

### Morphological analysis

After the appropriate time of Ro treatment (or DMSO in controls) in culture, 1.5 × 10^5^ MLL-AF9 BM or human leukemia cells (K562 and MOLM13) were washed once with 1× PBS, counted and centrifuged onto slides for 5 minutes at 35 × *g* and air-dried for 24 h prior to Richard-Allan Scientific Three-Step Stain Staining Set (Thermo Scientific) based on Eosin Y and Methylene Blue/ Azure A and mounted with Permount solution (Fisher). Cell morphology was evaluated by light microscopy at ×400 magnification (Zeiss Imager M-2 equipped with AxioCam ERc 5 s).

### Apoptosis measurements

Apoptosis measurements were taken by MUSE^TM^ Cell Analyzer (Millipore) using the MUSE^TM^ Annexin V and Dead Cell Assay Kit (Millipore) as recommended by the instructions from the manufacturer. Dot plots showing viability versus Annexin V + cells are shown in Supplementary Figs. 5 and 6.

### In vivo MLL-AF9 leukemia model and Ro 08–2750 administration

A total of 10,000 of MLL-AF9 BM secondary mouse leukemia cells previously obtained^7^ were injected retro-orbitally into female C57BL/6 (10–12-weeks-old) recipient mice that had been sublethally irradiated at 475 cGy. Drug administration (Ro 08–2750, 13.75 mg kg^−1^, DMSO) was performed by intraperitoneal injections (50 μL, top tolerated DMSO volume) 3 weeks after BM transplants (when showing signs of disease) for pharmacodynamic experiments (Fig. 6a), and 3 days after BM transplant for in vivo long-term studies (Fig. 6d). Mice weight was monitored every day to check for toxicity. All animal studies were performed on animal protocols approved by the Institutional Animal Care and Use Committee (IACUC) at Memorial Sloan Kettering Cancer Center.

### Fluorescence polarization

To validate RNA-binding activity inhibition by Ro 08–2750 and derivatives (Ro-OH, Ro-NGF) we used Fluorescence Polarization (FP) based assay in 384-well format for dose-response curve studies^36^. The RNA oligo used (Cy3-C_9_[spacer]-rGUAGUAGU, Integrated IDT Technologies) contained 2 MSI motifs (G**UAG**U) and had 8-nucleotides of length, optimal to minimize background and unspecific interactions. Here, manual pipetting was used to plate the reagents and the FP reading was performed in a BioTek Synergy Neon Plate Reader (High-Throughput Screening Resource Center, HTSRC, Rockefeller University).

### Microscale thermophoresis

For binding affinity studies of RNA and small-molecules to proteins of interest, purified recombinant GST-MSI2 WT, K22, F66, F97 and R100 to single alanine (A), triple (F66A/F97A/R100A) mutants, GST-RBP controls (SYNCRIP, SRSF2, HUR, RBMX, TIA-1) and bovine serum ALBUMIN were NT647-labeled using an amine-coupling kit (NanoTemper Technologies). Runs were performed at a concentration range of 50–120 nM (MSI2 and mutants) and 60 nM (SYNCRIP) to get optimal fluorescence signal using an LED power of 40–50% in a red laser equipped Monolith NT.115 (NanoTemper Technologies) (HTSRC, Rockefeller University). Prior to each run, protein preparations were diluted in MST buffer (50 mM HEPES, 100 mM NaCl, 0.05% Tween-20, pH 7.4) and protein aggregation was minimized by centrifuging the solutions at 20,820×*g* for 10 min. GST-proteins or GST-protein/RNA complexes (15 min pre-incubation) were mixed with increasing concentrations of small-molecules (0.015 to 500 μM) or RNA (0.0015 to 50 μM) and loaded onto 16 Premium Coated capillaries. The RNA oligo used (rGUAGUAGUAGUAGUA, Integrated IDT Technologies) contained 4 MSI motifs (G**UAG**U) and was 15-nucleotides long. The MicroScale Thermophoresis (MST) measurements were taken at RT and a fixed IR-laser power of 40% and 20 s per capillary. GraphPad Prism was used to fit the normalized data and determine apparent *K*_D_ values, represented as percent of fraction bound.

### Isothermal titration calorimetry

Due to incompatible fluorescence interference of labeled-RNA in MST assay, we used a non-labeled RNA probe of 15-nucleotides (15-nt, rGUAGUAGUAGUAGUA, Integrated IDT Technologies), poly(A) RNA (Sigma-Aldrich) and recombinant GST-MSI2 to assess direct Ro interaction with these agents. 15-nt or poly(A) RNA (1 mM stock in RNAse free H_2_O) were diluted to a 10 μM final concentration with Isothermal Titration Calorimetry (ITC) buffer (10 mM HEPES + 10% 10 mM Citrate Phosphate and 0.05% Tween-20, pH 7.0) and was titrated against 100 μM Ro 08–2750 or palmitate [positive poly(A) RNA control] in the same buffer by using MicroCal PEAQ-ITC range (Malvern Panalytical, HTRSC, Rockefeller University). As a protein binding control and to confirm the binding affinity (*K*_D_) obtained by MST, we titrated GST-MSI2 (full-length) at 30 μM in ITC buffer against 100 μM and 300 μM Ro 08–2750 obtaining similar *K*_D_ values (see Supplementary Fig. 1e for 100 μM). Kinetic and thermodynamic parameters were analyzed and fitted by AFFINmeter web-based software (www.affinimeter.com) and final graphs were represented by GraphPad Prism v7.0.

### Chemiluminescent electrophoresis mobility shift assays

An Electrophoresis Mobility Shift Assay (EMSA) approach to assess MSI2-RNA complexes and the inhibitory effect of small-molecules was set up by using LightShift Chemiluminescent RNA EMSA kit (Thermo Scientific). In brief, GST-MSI2 (125–250 ng) was preincubated with DMSO or the small-molecule (typically 20 μM final concentration) during 1 h at RT in 1X RNA EMSA binding buffer (10 mM HEPES, 20 mM KCl, 1 mM MgCl_2_, 1 mM DTT, Thermo Scientific) supplemented with 5% glycerol, 100 μg mL^−1^ tRNA and additional 10 mM KCl. After this period, 40 nM of biotinylated-RNA (biotin-rGUAGUAGUAGUAGUA, Integrated IDT Technologies) was added to the mixture (20 μL final volume) and incubated 1 h at RT. During this second incubation period, a 4–20% TBE polyacrylamide gel (BioRad) was pre-run at 100 V for 30–45 min in cold 0.5X TBE (RNAse free). Five microliter of 5× loading buffer was added to the 20 μL reaction and loaded into the pre-run TBE gel and voltage set at 100 V. Samples were electrophoresed until 3/4 of the length of the gel. Samples were then transferred in 0.5X TBE at 350–400 mA for 40 min. Membranes were then crosslinked with UV-light crosslinking instrument (UV Stratagene 1800) using Auto-Cross Link function. Membranes were either stored dry for development next day or developed using the detection biotin-labeled RNA chemiluminescence kit (as indicated by the manufacturer) (Thermo Scientific) and Hyperfilm ECL (GE Healthcare).

### Cloning, expression, and purification of GST tagged proteins

Human full-length MSI2 was cloned into the retroviral backbone pMSCV-IRES-BFP (MIB) vector (a gift from Dario Vignali; Addgene plasmid #52115) by Custom DNA Constructs (University Heights, Ohio) introducing a 5′Flag tag (5′-ATGGATTACAAGGATGACGACGATAAG-3′) and using BamHI and EcoRI restriction sites. Human full-length MSI2 was cloned into pGEX6P3^36^. RNA-recognition motif 1 (RRM1) from human MSI2 (nucleotides 64–270, NM_138962.2) was subcloned into empty pGEX6P3 using EcoRI and NotI restriction sites. Human SYNCRIP (hnRNP-Q variant 3, NM_001159674.1) was subcloned into empty pGEX6P3 (GE Healthcare) by introducing a 5′-Flag sequence and using SalI and NotI sites. GST-Flag-MSI2 wild-type (WT), Flag-MSI2 mutants, GST-RRM1 and GST-Flag-SYNCRIP recombinant proteins were produced in BL21 (DE3) competent cells (Agilent Technologies, Santa Clara, CA) as reported for MSI2 WT^36^. Here, GST-SYNCRIP protein required higher content of NaCl (250 mM) in the 1× PBS dialysis step and final buffer for optimal storage and performance in the biochemical and biophysical assays. Human RNA binding motif protein, X-linked (RBMX), transcript variant 1 (NM_002139, Myc-Flag-tagged, #RC200777, OriGene) cDNA was subcloned into pGEX6P3 by using AseI and SalI sites and purified under same conditions as GST-MSI2. The rest of RRM-based RBPs used in MST were commercially available as GST-tag purified recombinant proteins: human HUR (ELAVL1, 1–100, #H00001994-Q01), human TIA-1 (#H00007072-P01) and human SRSF2 (#H00006427-P01) all from Abnova (Taipei City, Taiwan). All the generated constructs are available upon request.

### Site-directed mutagenesis

To perform site-directed mutagenesis into pGEX6P3-Flag-MSI2 and pMSCV-IRES-BFP(MIB)-MSI2 constructs and obtain the corresponding recombinant GST-MSI2 mutants or express the MSI2 mutants in MLL-AF9 Puro-CreER + Msi1^*fl/fl*^ Msi2^*fl/fl*^ bone marrow cells, we used QuikChange Lightning and Multi Site-Directed Mutagenesis Kit (#210513 and #210518, Agilent Technologies). The primers were designed for full-length pGEX6P3-Flag-MSI2 and pMIB-MSI2 constructs using QuickChange Primer Design (https://www.genomics.agilent.com/primerDesignProgram.jsp) and were the following: K22A (Fwd: 5′-GTCCACCGATAAACATTGCACCGGGGTCGTGCTGGG-3′; Rev: 5′-CCCAGCACGACCCCGGTGCAATGTTTATCGGTGGAC-3′), F66A (Fwd: 5′-GCTCCAGAGGCTTCGGTGCCGTCACGTTCGCAG-3′, Rev: 5′-CTGCGAACGTGACGGCACCGAAGCCTCTGGAGC-3)′; F97A (Fwd: 5′-AGACGATTGACCCCAAAGTTGCAGCTCCTCGTGCAGCGCAACCCAA-3′, Rev: 5′-TTGGGTTGCGCTGCACGAGGAGCTGCAACTTTGGGGTCAATCGTCT-3′) and R100A (Fwd: 5′-CCAAAGTTGCAGCTCCTCGTGCAGCGCAACCCA-3′, Rev: 5′-TGGGTTGCGCTGCACGAGGAGCTGCAACTTTGG-3′). PCR reactions and cloning were perfomed as indicated by the manufacturer (Agilent Technologies) with modified annealing temperatures according to primer *T*_m_ values. All these mutation plasmids are available upon request.

### Human MSI2 RRM1 recombinant protein production

GST-RRM1 protein was initially produced in BL21 (DE3) competent cells (Agilent Technologies, Santa Clara, CA) as previously reported for MSI2 WT^36^. Here, the cell lysate of 4 L initial culture was centrifuged at 22,640×*g* for 1 h and the resulting volume applied to a XK16/20 column pre-packed with Glutathione Sepharose 4 Fast Flow connected to an AKTA Prime FPLC (GE Healthcare). To obtain the RRM1 optimal prep for the crystal preparation, the collected fractions containing GST-RRM1 (50 mM Tris-HCl, 20 mM reduced L-Glutathione, pH 8.0) were pooled and dialyzed against PreScission Protease Buffer (50 mM Tris-HCl, 150 mM NaCl, 1 mM EDTA, 1 mM DTT, pH 7.5). GST tag was then cleaved with PreScission Protease overnight at 4 °C. Pure RRM1 fractions were obtained through size exclusion chromatography (HiLoad Superdex 75, GE Healthcare) and concentrated with a 3 K Amicon Ultra Centricon (Millipore).

### Crystallization and structure determination

A final concentrated MSI2 RRM1 pure protein preparation (>98% by coomassie) at 2 mg mL^−1^ in 50 mM Tris-HCl, pH 7.5 was crystallized by sitting drop vapor diffusion. A 1 μL of protein solution was mixed with an equal volume of precipitant solution containing 100 mM Tris, 200 mM Li_2_SO_4_, 25% PEG 3350 (pH 8.5). Crystals appeared after two weeks. They were cryoprotected by mother liquor containing 25% glycerol and flash frozen in liquid nitrogen. X-ray diffraction data were collected from single crystals at the Advanced Photon Source beamline 24ID-C. The temperature was 100 K and the wavelength was 0.9792 Å. Indexing and merging of the diffraction data were performed in HKL2000^52^. The phases were obtained by molecular replacement by PHENIX^53^ using PDB entry 1UAW as the search model. Interactive model building was performed using O^54^. Refinement was accomplished with PHENIX. Data collection and refinement statistics are summarized in Table 1. Ramachandran statistics were used, 100% of the residues are in the favored regions of Ramachandran plot. The crystal structure has been deposited in RCSB PDB under the accession code 6DBP.

### RRM1 protein expression and NMR spectroscopy

The sequence of RRM1 (nucleotides 64–270, NM_138962.2) was cloned into a pRSFDuet-1 vector (Novagen) engineered with an N-terminal hexahistidine (His6)-SUMO tag. The fusion protein was expressed in *E. coli* strain BL21-CodonPlus(DE3)-RIL (Stratagene). For uniform ^15^N/^13^C isotopic enrichment of the protein, bacteria cells were grown in M9-minimal medium containing ^15^NH_4_Cl (Cambridge Isotope Laboratories) and [^13^C_6_]-glucose as sole nitrogen and carbon source, respectively, supplemented with 50 μg ml^-1^ kanamycin at 37 °C to OD_600_ of 0.8, and induced by 1 mM isopropyl β-D-1-thiogalactopyranoside (IPTG) at 18 °C overnight. Bacteria cells were harvested by centrifugation at 4 °C and lysed by the EmulsiFlex-C3 homogenizer (Avestin) in buffer A (50 mM Tris-HCl, 500 mM NaCl and 20 mM imidazole, pH 8.0) supplemented with 1 mM phenylmethylsulfonyl fluoride (PMSF) protease inhibitor and 0.5% Triton X-100. Cell lysates were centrifuged at 48,300×*g* for 0.5 h in a JA-20 fixed angle rotor (Avanti J-E series centrifuge, Beckman Coulter). The supernatant was loaded onto a 5 mL HisTrap FF column (GE Healthcare), and followed by extensive washing with buffer A. The target protein was eluted with buffer A supplemented with 400 mM imidazole. The elution protein was incubated with ubiquitin-like protease (ULP1) during dialysis at 4 °C overnight against buffer B containing 20 mM Tris-HCl, pH 7.5, 20 mM imidazole, 100 mM NaCl, 1 mM MgCl_2_, and 5 mM β-mercaptoethanol. The SUMO-tags were removed by reloading on HisTrap FF column. The flow-through was further loaded on 5 mL HiTrap Heparin column (GE Healthcare) pre-equilibrated in buffer B. Elution of recombinant proteins was achieved by a linear gradient from 100 mM to 1 M NaCl in 15 column volumes. The fractions were monitored by SDS-PAGE, and target proteins were concentrated by Amicon concentrators. The sample was loaded on Superdex 200 16/60 column pre-equilibrated in buffer C (20 mM MES, 150 mM NaCl, pH 6.0). The high purity eluting fractions were detected by SDS-PAGE and collected. Protein concentrations ranged from 100 μM ^1^H/^15^N and 140 μM ^1^H/^13^C/^15^N labeled RRM1 in NMR buffer (buffer C dissolved in 90% H_2_O/10% D_2_O). The protein was flash-frozen in liquid nitrogen and stored at −80 °C.

The NMR data was acquired on Bruker *AVANCE* series of spectrometers equipped with *Z*-axis gradient TCI/TXI CryoProbes^TM^ at a sample temperature of 5 °C and B_o_ field strengths of 500.13 and 800.23 MHz, respectively. To map the binding site from chemical shift perturbations the stock solution of the compound was titrated into 100 μM ^1^H/^15^N-labeled RRM1 at five different protein to compound ratios (1:2, 1:4, 1:6, 1:8, 1:10). The backbone resonances of ^1^H/^13^C/^15^N labeled RRM1 in the presence (1:14 ratio) and absence of the compound Ro 08–2750 were assigned at 5 °C using a standard suite of triple resonance experiments^55^, HNCO, HNCA, HNCACB and CBCA(CO)NH acquired at 800 MHz. The multidimensional NMR datasets were processed in Topspin 2.1 from Bruker Biospin and the chemical shifts analyzed in CARA1.5^56^.

### RNA purification and quantitative real-time PCR

Total RNA was isolated from 1–2 × 10^6^ cells dry pellets kept at −80 °C for less than a week using QIAgen RNeasy Plus Mini kit. cDNA was generated from RNA using iScript cDNA Synthesis (#1708891, BioRad) with random hexamers according to the manufacturer’s instructions. Real-time PCR reactions were performed using a Vii7 sequence detection system. Relative quantification of the genes was performed using Power SYBR Mix (2×) and specific primers for human *c-MYC* (Fwd: 5′-TCAAGAGGCGAACACACAAC-3′, Rev: 5′-GGCCTTTTCATTGTTTTCCA-3′), *TGFβR1* (Fwd, 5′-GCACAACAAAATCACTATCCCATTAG-3′, Rev, 5′-CATTTGGAGCCAGAACACTGC-3′), *SMAD3* (Fwd, 5′-CAGCTGTGTCTGCCAAACACA-3′, Rev, 5′-GGCCGGTGGTGTAATACTACCTG-3′), *HOXA9* (Fwd, 5′-CAGACCCTGGAACTGGAGAA-3′, 5′-ATTTTCATCCTGCGGTTCTG-3′) and *CDKN1A* (Fwd, 5′-CCTCATCCCGTGTTCTCCTTT-3′, Rev, 5′-GTACCACCCAGCGGACAAGT-3′) and the 2^−ΔΔ*C*t^ method as described by the manufacturer using *β*-*ACTIN* (Fwd, 5′-AAACTGGAACGGTGAAGGTG-3′, Rev, 5′-AGAGAAGTGGGGTGGCTTTT-3′) to normalize data.

### Immunoblot analysis

For immunoblot analysis, Ro treated and DMSO control MOLM13 or K562 cells (routinely at 0.5 × 10^6^ cells mL^−1^) were counted and washed twice with cold PBS before collection. 1–5 × 10^6^ cells were resuspended and lysed in 250 μl of 1× RIPA Buffer supplemented with Protease Inhibitor Tablets (Sigma-Aldrich) buffer for 30 min on ice. After centrifugation at 20,820 × *g* on a top-bench centrifuge, lysate (supernatant) was collected and total protein quantified by BCA (Thermo Scientific). Cell lysates were separated by 4–15% SDS–PAGE and transferred to 0.45 μm nitrocellulose membrane. Membranes were blocked and were blotted overnight (4 °C) for TGβR1 (ab31013, Abcam, 1:750 dilution), SMAD3 (9523S, Cell Signaling Technology, 1:750 dilution), HOXA9 (07–178, Millipore, for drug dose-dependent experiments and ab140631, Abcam; 1:1000 dilution for time-course experiments), c-MYC (5605, Cell Signaling Technology; 1:1,000 dilution), P21 (2947 S, Cell Signaling Technology, 1:750 dilution), MSI2 (ab76148, Abcam; 1:2,000 dilution) and β-ACTIN-HRP conjugated (A3854, Sigma-Aldrich; 1:20,000 dilution) and developed by Hyperfilm ECL (GE Healthcare) with ECL and pico-ECL reagents (Thermo Scientific). Uncropped and unprocessed scans are included in the Source Data file.

### Luminescence-based cytotoxicity assays

A total of 10,000 cells (MLL-AF9+ BM from secondary transplants or human leukemic cell lines -K562 or MOLM13-) were platted into U-bottom 96-well plates in the presence of increasing concentration of small-molecules (Ro, Ro-OH or Ro-NGF) up to 100 μM (in 1:2 serial dilutions). Cells were cultured for 72 h at 37 C in a 5% CO_2_ incubator. To read cell viability, Cell-Titer Glo kit (Promega) was used. After cooling down cells to RT for 20–30 min, 100 μL of the cultured cells were transferred to opaque-white bottom 96-well plates and mixed with 100 μL of Cell-Titer Glo reagent. The mixture was incubated for 15 min at RT and read using a Synergy H1 Hybrid reader (BioTek) for luminescence. Data was normalized as percentage viability and graphed by non-linear regression curves in Graph Pad PRISM 7.0.

### RNA immunoprecipitation

To assess mRNA enrichment and blocking of protein-binding to mRNA by the small-molecules we performed RNA immunoprecipitation (RNA IP) experiments using Magna RIP RNA-binding protein immunoprecipitation kit (#03–115, Millipore). 25 × 10^6^ K562-MIG or MSI2 overexpressing cells 1 h treated with DMSO (control) or Ro μM were used. First, cells were washed with cold PBS and lysed. Five micrograms of mouse anti-Flag (clone M2, #F1804, Sigma-Aldrich) antibody incubated with magnetic beads were used to immunoprecipitate Flag-MSI2 K562 cells. After washing the immunoprecipitated, they were treated with proteinase K. RNA extraction was performed by the phenol–chloroform method, and 200–500 ng of purified RNA was converted to cDNA using the Verso cDNA kit (Thermo Scientific). qPCR was used to validate target mRNAs bound by MSI2 and control cells.

### *O*-Propargyl-Puromycin incorporation by flow cytometry

Cells were plated at a density of 200,000 cells mL^−1^ and pre-treated with DMSO or Ro up to 4 h. Then, 50 μM *O*-propargyl-puromycin (OP-Puro; NU-931–05, Jena Bioscience) was added. Control cells were co-incubated with DMSO or Ro and treated with 150 μg mL^-1^ cycloheximide for 15 min. Non-OP-Puro treated cells were also used as negative controls for flow cytometry. Cells were washed twice before collection and subjected to processing using the Click-iT Flow Cytometry Assay kit (#C10418, Invitrogen) following the manufacturer’s instructions. Labeled cells were analyzed using a BD LSR Fortessa instrument and graphed as Alexa Fluor 647 (AF647) Mean Fluorescence Intensity (normalized to DMSO control treated with OP-Puro).

### RNA sequencing

Total RNA was isolated from 1 × 10^6^ dry pellets of K562 and MOLM13 4 h treated with DMSO (control) or Ro 20 μM (*n* = 4 for each group) using Qiagen RNeasy Plus Mini kit and the quality assessed on a TapeStation 2200 (Agilent technologies). QuantSeq 3′ mRNA-Seq Library Prep Kit FWD (Lexogen, Vienna Austria), supplemented with a common set of external RNA controls, according to manufacturer’s recommendations (ERCC RNA Spike-In mix, ThermoFisher Scientific, #4456740). An in-house pipeline was used for read mapping and alignment, transcript construction and quantification of data generated by sequencing (HiSeq 2000, NYGC, NY, USA). This procedure was done in the Epigenetics Core from MSKCC. RNA-seq data have been deposited in NCBI GEO database with the accession code GSE114320.

### Synthesis of Ro-OH by reduction of Ro 08–2750 aldehyde

To a cooled (0 °C) slurry of Ro 08–2750 (19 mg, 0.070 mmol) in anhydrous MeOH (1.9 mL) was added LiBH_4_ (32 mg, 1.5 mmol) in portions over 5 min. The slurry turned from bright orange to dark brown, then dark green within 10 min. The reaction mixture was removed from the ice bath and allowed to warm to room temp (22 °C) over 2 h. Reaction progress was monitored by LC-MS (5–95% MeCN in H_2_O). Four portions of LiBH_4_ (10 mg, 0.04 mmol) were added every 12 h until the reaction was complete. The reaction was quenched with AcOH (10 mL) and filtered. The solids were washed with water (5 mL), MeOH (5 mL), and Et_2_O (5 mL). The solid was collected and dried under vacuum to provide a pale orange solid (7 mg, 26%). Purification by HPLC (5–95% MeCN in H_2_O) afforded the product as an orange solid (3 mg, 16%). The synthesis was adapted from Salach et al.^57^.

^**1**^**H-NMR** (600 MHz, DMSO) δ 11.34 (s, 1 H), 7.91 (s, 1 H), 7.87 (s, 1 H), 5.69 (t, *J* *=* 4.5, 1 H), 4.74 (d, *J* *=* 4.4, 2 H), 3.99 (s, 3 H), 2.38 (s, 3 H). ^**13**^**C-NMR** (150 MHz, DMSO) 159.8 (C), 155.4 (C), 150.5 (C), 149.7 (C), 137.4 (C), 133.57 (C), 133.56 (C), 131.5 (CH), 131.0 (C), 112.3 (CH), 60.8 (CH_2_), 31.7 (CH_3_), 17.2 (CH_3_); IR (ATR): 2361, 2341, 1717. **ESI-MS** *m/z* (rel int): (pos) 273.1 ([M + H]^+^, 100).

### Statistical analysis

Student’s *t* test was used for significance testing in the bar graphs, except where stated otherwise. A two-sample equal-variance model assuming normal distribution was used. The investigators were not blinded to the sample groups for all experiments. *P* values less than 0.05 were considered to be significant. Graphs and error bars reflect means +/− standard error of the mean except stated otherwise. All statistical analyses were carried out using GraphPad Prism 7.0 and the R statistical environment.

### Modeling and system preparation for computational modeling

System preparation, modeling, and initial docking calculations were performed using the Schrödinger Suite molecular modeling package (version 2015–4), using default parameters unless otherwise noted. The MSI2 RRM1 protein structure (PDB ID: 6DBP) was prepared using the Protein Preparation Wizard^58^. In this step, force field atom types and bond orders were assigned, missing atoms were added, tautomer/ionization states were assigned, water orientations were sampled, and ionizable residues (Asn, Gln, and His residues) have their tautomers adjusted to optimize the hydrogen bond network. A constrained energy minimization was then performed. All crystallographically resolved water molecules were retained.

Potential binding sites were explored and characterized using the SiteMap^59^ tool. Ligands with experimental activity and known inactives were docked into putative binding sites using Glide SP^60,61^ to evaluate enrichment of known actives. Best docking scores were for the ‘Ro’ series for the ‘(−)-gossypol’ binding site described by Lan et al.^43^ compared with other putative pockets.

Since the receptor may not be in an optimal conformation to bind small molecule inhibitors, induced fit docking^62^ of ligand Ro 08–2750 was performed to this binding pocket. Induced fit docking results were validated with the metadynamics protocol described by Clark et al.^63^. In these metadynamics simulations a biasing potential is applied to the ligand RMSD as collective variable. The resulting potential energy surface is evaluated towards how easy a ligand can move away from the initial binding mode. The underlying assumption is that a ligand pose which is closer to the real one has a higher energetic barrier to leave the pose than an incorrect pose. The pose ranked second using the induced fit docking score retrieved the best score from the metadynamics ranking protocol compared with the other induced fit docking poses. This receptor configuration was furthermore tested towards its suitability for a virtual screening by a Glide SP docking of known actives into this pocket. The docking scores using this receptor conformations were better (down to −6.2) compared with the initial protein conformation in the crystal structure. Furthermore, a WaterMap^64^ calculation was done for this receptor.

### Induced fit docking of Ro-NGF and Ro-OH compounds

Induced Fit Docking (IFD) was performed against the receptor pose from the selected Ro 08–2750 pose, using Schödinger molecular modeling suite (version 2017–4). Poses for Ro-NGF and Ro-OH, the top and second scored poses, respectively, were selected to most closely match the Ro 08–2750 pose.

### Alchemical free energy calculations

Absolute alchemical free energy calculations were carried out to validate the putative binding poses in a fully flexible explicitly solvated system. The YANK GPU-accelerated free energy calculation code with the Amber family of forcefields was used for this purpose. Details follow:

System preparation and modeling: the top poses generated by induced fit docking, as described above, were selected as input protein and ligand poses. Because proteins and ligands were already prepared, they were simply run through the pdbfixer 1.4 command line tool with add-atoms and add-residues set to None to convert residue and atom names to be compatible with Amber tleap.

Parameterization: tleap (from the minimal conda-installable AmberTools 16 suite ambermini 16.16.0) was used to solvate the complex in a cubic box with a 12 Å buffer of TIP3P water molecules around the protein. The system was parameterized using AMBER’s forcefield ff14sb^65^ and GAFF 1.8^66^. Missing ligand parameters were determined using antechamber^67^. The ligand was assigned charges using the AM1-BCC^68^ implementation in OpenEye (OEtoolkit 2017.6.1 through openmoltools 0.8.1).

Minimization: minimization was performed using the implementation of the L-BFGS algorithm in OpenMM 7.1.1^69^ with a tolerance of 1 kJ mol^−1^nm^−1^.

Production Simulation: production simulation was run using YANK 0.19.4^70^ using OpenMMTools 0.13.4. In order to keep the ligand from diffusing away from the protein while in a weakly coupled state, it was confined to the binding site using a Harmonic restraint with an automatically determined force constant (*K* = 0.33 kcal mol^−1 ^Å^−2^). The restraint was centered on the following receptor residues using all-atom selection: 2, 4, 46, 76, 78, and 80. The ligand atoms were automatically determined. The calculation was performed using particle mesh Ewald (PME)^71^ electrostatics with default YANK settings with a real-space cutoff of 9 Å. A long-range isotropic dispersion correction was applied to correct for truncation of the Lennard-Jones potential at 9 Å. The system was automatically solvated with TIP3P^72^ solvent and four neutralizing Cl^−^ions, paramterized using the Joung and Cheaham parameters^73^. Production alchemical Hamiltonian exchange free energy calculations were carried out at 300 K and 1 atm using a Langevin integrator (VRORV splitting)^74^ with a 2 fs timestep, 5.0 ps^-1^ collision rate, and a molecular-scaling Monte Carlo barostat. Ro 08–2750 and Ro-NGF were run for 10,000 iterations (50 ns/replica) with 2500 timesteps (5 ps) per iteration, while Ro-OH was run for 15,000 iterations (75 ns/ replica) with 2500 timesteps (5 ps) per iteration. Complex configurations were stored for each replica once per iteration. Replica exchange steps were performed each iteration to mix replicas using the Gibbs sampling scheme^75,76^. The alchemical pathway was automatically determined for each compound using the YANK autoprotocol protocol trailblazing feature.

Absolute binding free energy estimates: absolute free energies (*ΔG*) of binding for each compound was estimated using MBAR. Samples were reweighted to a cutoff of 16 Å to correct the isotropic dispersion correction to a non^−^isotropic long-range dispersion. This correction is important to account for the heterogeneous density of protein. To remove the harmonic restraint bias, samples were reweighted to substitute a squared well restraint of radius 10 Å.

Clustering analysis: the fully interacting trajectory from YANK was extracted to a PDB file, discarding the following number of initial iterations, which came prior to equilibration:^77^ 1500 for Ro 08–2750, 1600 for Ro-OH, and 1600 for Ro-NGF. These trajectories were aligned in MDTraj^78^ using only protein backbone atoms. The small molecules were then sliced out and clustered on Cartesian coordinates using the MSMBuilder^79^ implementation of RegularSpatial clustering using a 1 Å RMSD cutoff. For the most populated clusters for Ro 08–2750 and Ro-OH, cluster centers were selected and shown with 10 randomly sampled cluster members. Ro-NGF produced a large number of lowly populated clusters with highly heterogeneous binding poses, and were therefore not shown.

Conformational heterogeneity analysis: to investigate the conformational heterogeneity in the presence or absence of the ligand, the fully interacting thermodynamic state (corresponding to the holo protein bound to the ligand) and fully non-interacting state (corresponding to the apo protein free of ligand interactions) for all three ligands were extracted using a 4-frame skip, discarding the initial frames as above.

### Reporting summary

Further information on research design is available in the Nature Research Reporting Summary linked to this article.

## Supplementary information

Supplementary Information

Peer Review File

Reporting Summary

Description of Additional Supplementary Files

Supplementary Data

Source Data

## Data Availability

A reporting summary for this Article is available as a Supplementary Information file. Coordinates and structure factors have been deposited in the RCSB Protein Data Bank (PDB), under the accession code 6DBP. RNA-seq data have been deposited in NCBI Gene Expression Omnibus (GEO) database with the accession code GSE114320. The source data underlying Figs. 1b, 1d, 2d–j, 3f, 5e–h and Supplementary Figs. 1c–e, 2b, 2d, 2 f, 5c and raw data are provided as a Source Data file. All data is available from the corresponding author upon reasonable request.

## Code availability

All Schrödinger project files, YANK simulation inputs, and analysis scripts have been made publicly available (https://github.com/choderalab/musashi).
