# Supplementary material for: Small-molecule targeting of MUSASHI RNA-binding activity in acute myeloid leukemia
Source: Nat Commun. 2019 Jun 19;10:2691. doi: 10.1038/s41467-019-10523-3 (PMC6584500; doi:10.1038/s41467-019-10523-3)
Supplement: Supplementary file 1 — Supplementary Information [file 41467_2019_10523_MOESM1_ESM.pdf]

Supplementary Information. Supplementary Figures 1-8.

Minuesa et al. Small-molecule targeting of MUSASHI RNA-binding activity in acute myeloid leukemia

Supplementary Figure 1. Ro 08-2750 binds to RRM1 with conserved aminoacid residues and does not directly interact with RNA.

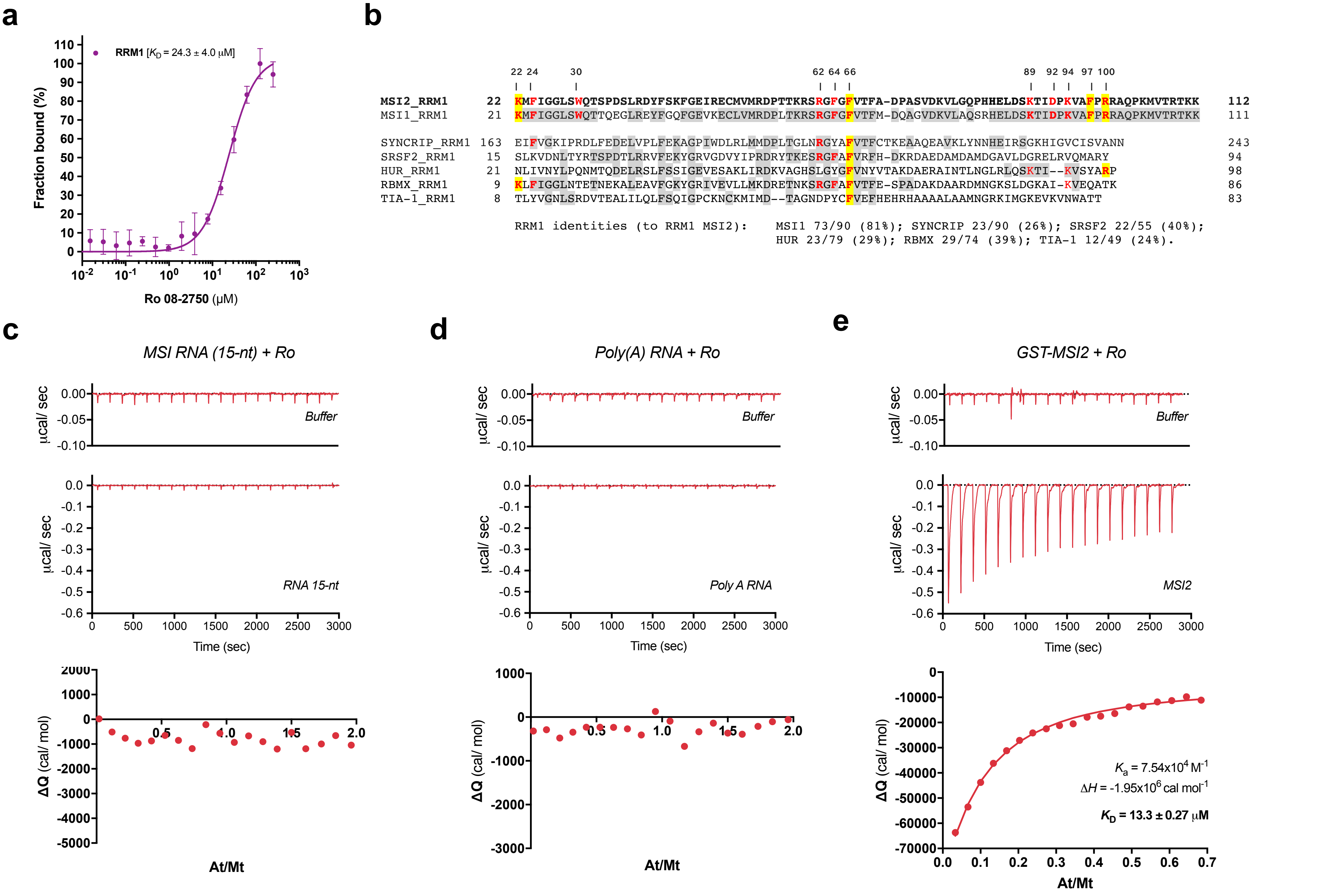

(a) MicroScale Thermophoresis (MST) assay showing interaction of Ro with GST-RRM1 (hMSI2). Ro concentrations ranged from 0.0153 to 500 μM. K_D values ± standard error mean (s.e.m.) (μM) of at least three experiments are shown as percentage of fraction bound. (b) Sequence alignment of RRM1 of human MSI2, MSI1 and RBP control proteins SYNCRIP, SRSF2, HUR, RBMX and TIA-1. Numbers indicate crucial RNA-binding conserved residues (in bold red) in hMSI2 (i.e. F24, corresponding to F23 in hMSI1, F165 in SYNCRIP). Grey highlights indicate conserved amino acids and yellow indicate conserved Ro interacting residues (i.e. F66 conserved in every RBP, F97 only in hMSI1). Isothermal Titration Calorimetry (ITC) assay assessing Ro affinity of interaction with MSI-motif containing RNA 15-nt (c), poly(A) RNA (d) and GST-MSI2 (d). Representative raw data for sequential injections (top two panels) of Ro into the RNAs (c, d) or recombinant protein (d) and integrated heat data after correction of the heat of dilution of Ro drug against buffer (bottom curves). The data (red circles) in (e) was fitted to a one-site model and the solid red line represent the best-fit of the data. Three experiments were run to confirm binding affinity and thermodynamic and affinity values shown in (e). Source data are provided as a Source Data file.

Supplementary Figure 2. Ro interacting residues in the RNA-binding site of RRM1 by docking and NMR analysis

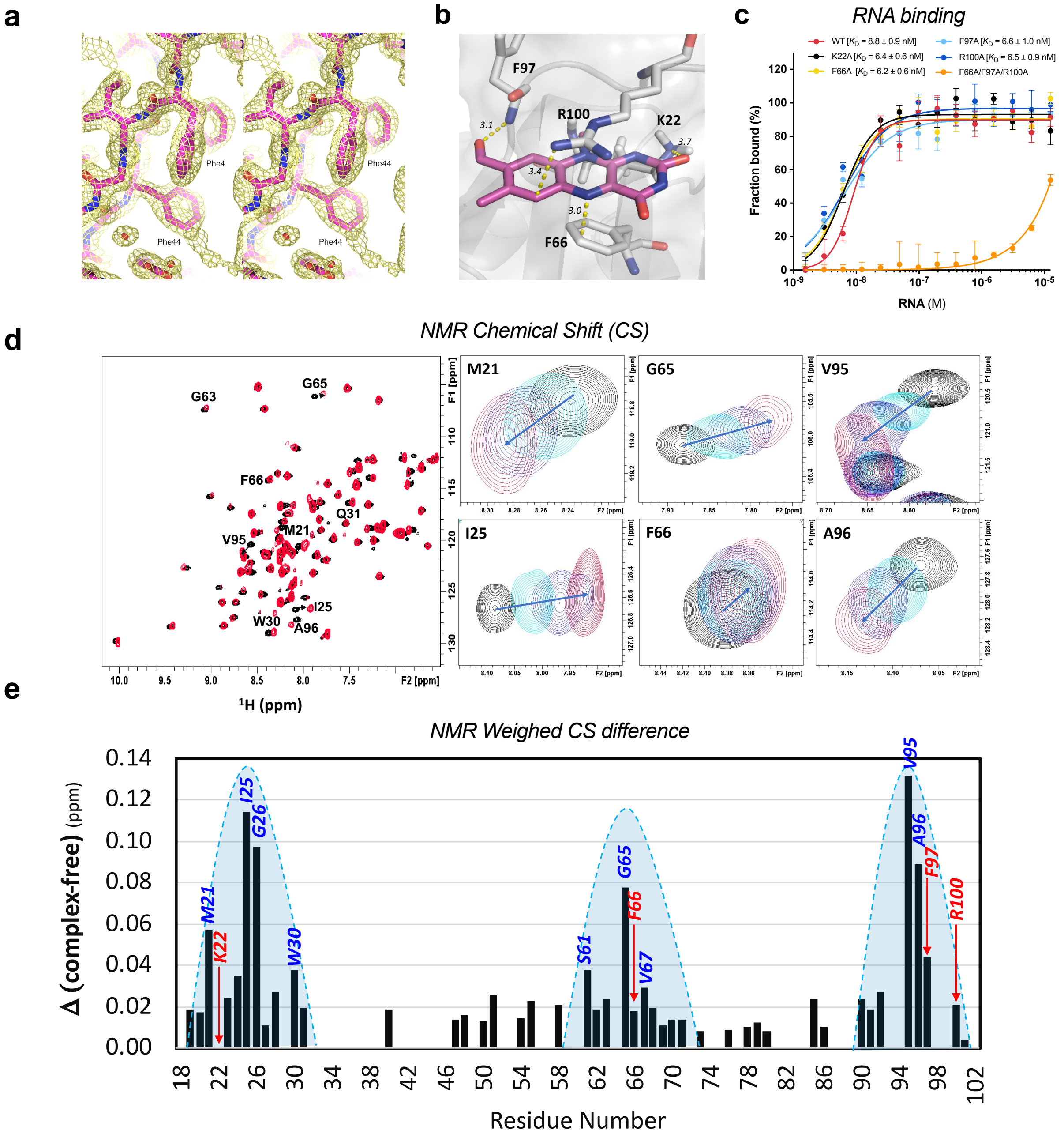

(a) A stereo view of the 2Fo-Fc electron density map in the area of RNA binding site, contoured at 1σ, is shown in yellow mesh. (b) Ro docking pose in the RNA-binding site of MSI2 RRM1 with interacting residues. Distances shown in Å; (c) MST experiments showing GST-MSI2 WT (*red*), K22A (*black*), F66A (*yellow*), F97A (*cyan*), R100A (*dark blue*) and Triple (F66A/F97A/R100A, *orange*) mutants interaction to MSI2 RNA oligo (4 MSI motifs; 15-nt). K_D values s.e.m. of at least three experiments are shown (nM); (d) Overlay of ^{15}N-HSQC spectrum of human RRM1 of MSI2 protein in the free (*black contours*) state and bound to excess of Ro compound (*red contours*, 1:8 ratio). The residues which show the largest chemical shift (CS) perturbation were annotated and the arrows indicate the direction of CS change through the titration. The right side panels display individual resonances from the ^{15}N-HSQC titration at different protein to compound ratios, black (1:0), cyan (1:2), blue (1:4) and red (1:8); (e) Histogram plot of weighted CS difference (Δ(complex-free) $\sqrt{[(\omega_H)^2 + (0.2^*\omega_N)^2]}$) calculated from the amide proton (ω_H) and nitrogen (ω_N) CS differences between the free and ligand bound protein complex at 1:8 ratio. The chemical shifts were measured at 500MHz and 5ºC. Source data are provided as a Source Data file.

Supplementary Figure 3. Synthesis, ^{1}H and ^{13}C NMR spectrum of Ro-OH

a

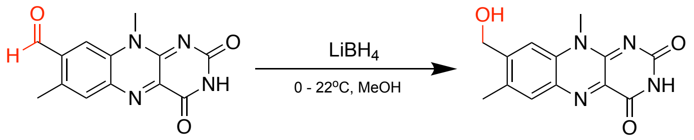

Ro **Ro-OH**

b

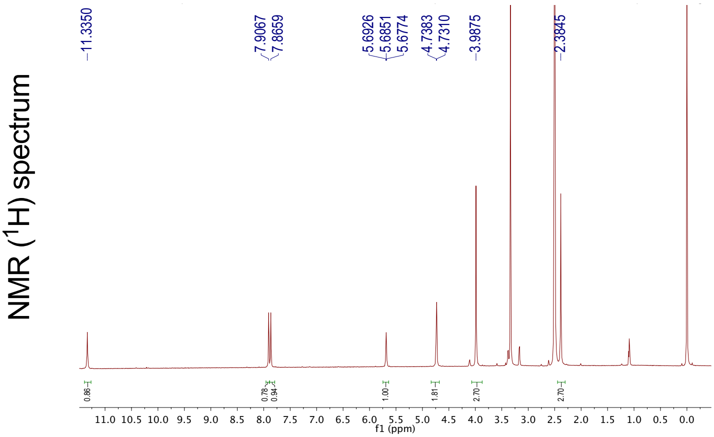

c

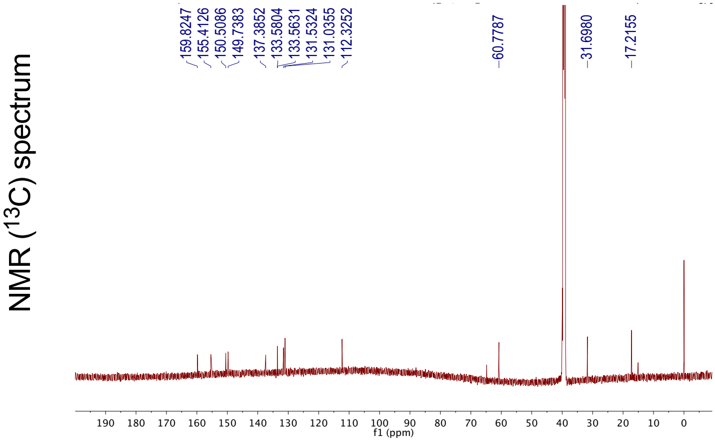

(a) Chemical synthesis of Ro-OH from Ro compound (see *Methods* for details).
(b) ^{1}H and (c) ^{13}C NMR séctrum of Ro-OH final product.

Supplementary Figure 4. Docking and alchemical free energy calculations identifies heterogeneous ensemble of binding poses.

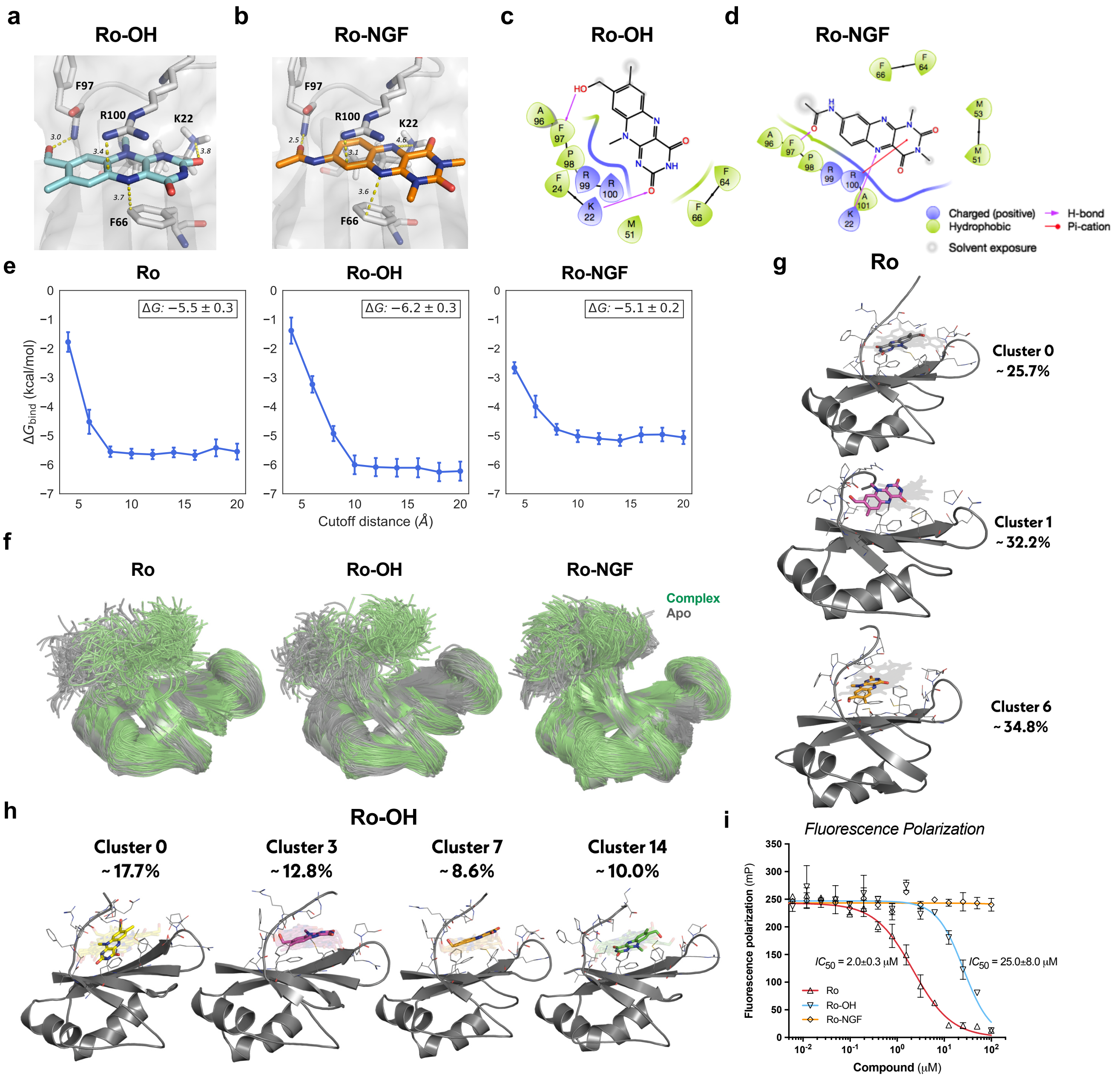

(a) Docked pose of Ro-OH in the RNA binding site of MSI2 RRM1. Distances in Å. (b) Docked pose of Ro-NGF in the RNA binding site of MSI2 RRM1 showing a displaced center of the small-molecule from the binding site. (c) 2D representation of Ro-OH docked pose in the RRM1 of MSI2. (d) 2D representation of Ro-NGF docked pose in the RRM1 of MSI2 showing H-bonding of K22 changing from the O to the N in the middle ring, and π-cation interacting with R100 displaced with respect to Ro (see Fig. 2b). Legend for 2D on the bottom right. (e) Computed binding free energy (ΔG_{bind}, kcal mol^{-1}) estimates from alchemical free energy calculations (*y-axis*) for Ro, Ro-OH, and Ro-NGF for different definitions of the "bound" complex as a function of distance cutoff (*x-axis*, in Å). Reported statistical errors and error bars correspond one standard error. The inset ΔG_{bind} was calculated for a cutoff of 20Å. (f) In the alchemical Hamiltonian replica exchange simulations, a conformational change is induced when MSI2 is bound ("Complex"; *green*) to Ro (*right*) or Ro-OH (*center*), as compared to apo MSI2 ("Apo"; *gray*). Ro-NGF (*left*) does not induce the same conformational change. (g) The top three most populous clusters for Ro. The protein structure and solid-color ligand pose depict cluster centers, while transparent ligand poses depict 10 randomly sampled frames assigned to that cluster. Sidechains within 4Å of any of the ligands are shown as lines. (h) The top four most populous clusters for Ro-OH. (i) FP confirmation of Ro, Ro-OH and Ro-NGF MSI2 RNA binding inhibition. IC_{50} values of two independent experiments performed in triplicate with s.e.m. are shown [IC_{50} = 2.0±0.3 μM (Ro, *red*), 25.0±8.0 μM (RoOH, *cyan*)]. Ro-NGF (*orange*) showed null inhibition of RNA-binding activity. Source data are provided as a Source Data file.

Supplementary Figure 5. Apoptosis and rescue colony forming assays in MLL-AF9 leukemic cells after treatment with Ro.

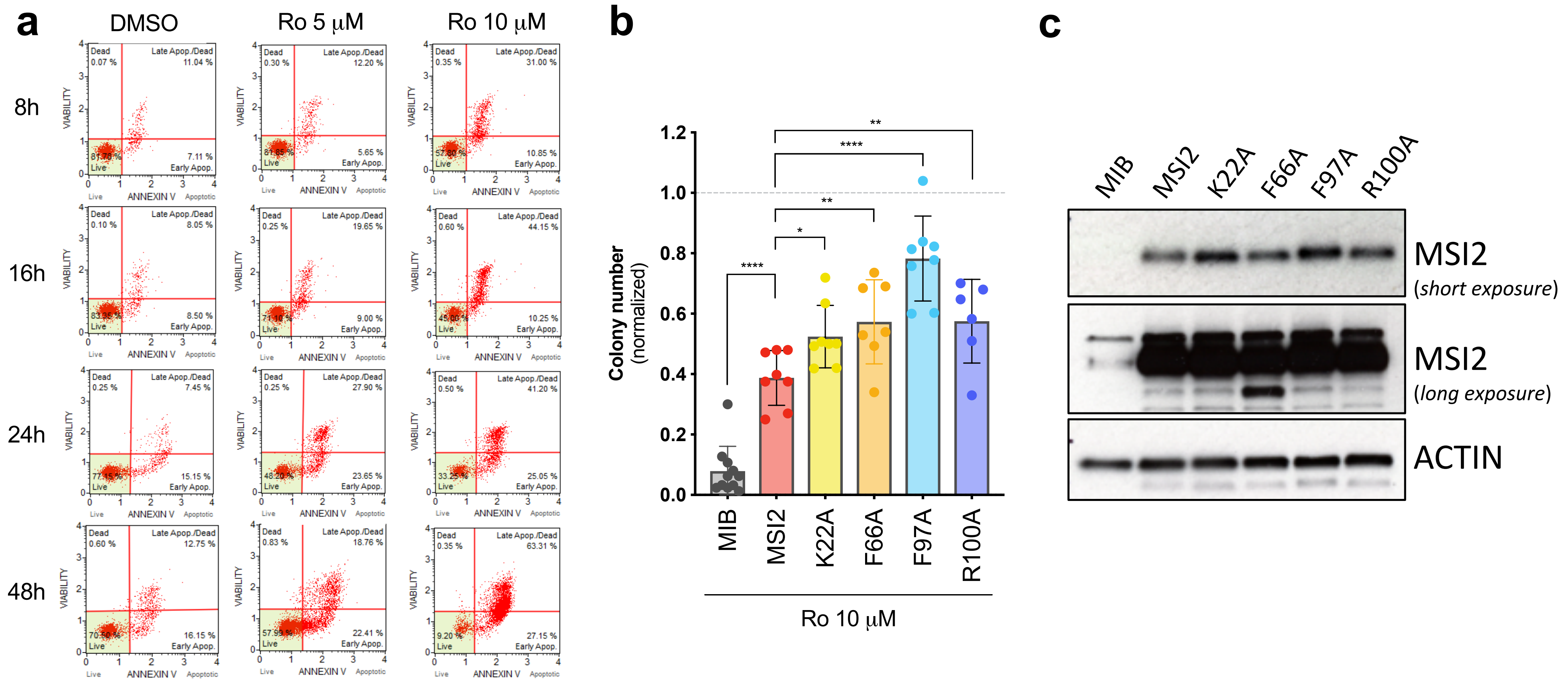

(a) Apoptosis plots (graphs in Fig. 3d) showing Annexin V+ and 7AAD (live/dead staining) by Apoptosis MUSE Cell kit and MUSE Cell Analyzer in MLL-AF9+ BM cells at 8, 16, 24 and 48 hours post treatment with Ro 5 and 10 µM. (b) CFU assay of MLL-AF9+ Puro-CreER+ Msi1$^{fl/fl}$ Msi2$^{fl/fl}$ BM MIB (empty vector, *grey*) and overexpressing MSI2 WT (*red*), K22A (*yellow*), F66A (*orange*), F97A (*cyan*) and R100A (*blue*) after Ro treatment at 10 µM. Results represent the average ± s.e.m. of normalized colony numbers (to MIB in DMSO: dashed grey line = 1.0) of at least four experiments performed in duplicate. Two tailed paired *t*-test (DMSO vs Ro treated); *p<0.05, **p<0.01, ****p<0.001. (c) Immunoblot analysis of MSI2 wild-type and mutants protein levels after retroviral overexpression experiments in MLL-AF9 BM cells used in panel (b). Source data are provided as a Source Data file.

Supplementary Figure 6. Differentiation and apoptosis are induced in MOLM13 and K562 cells after Ro treatment.

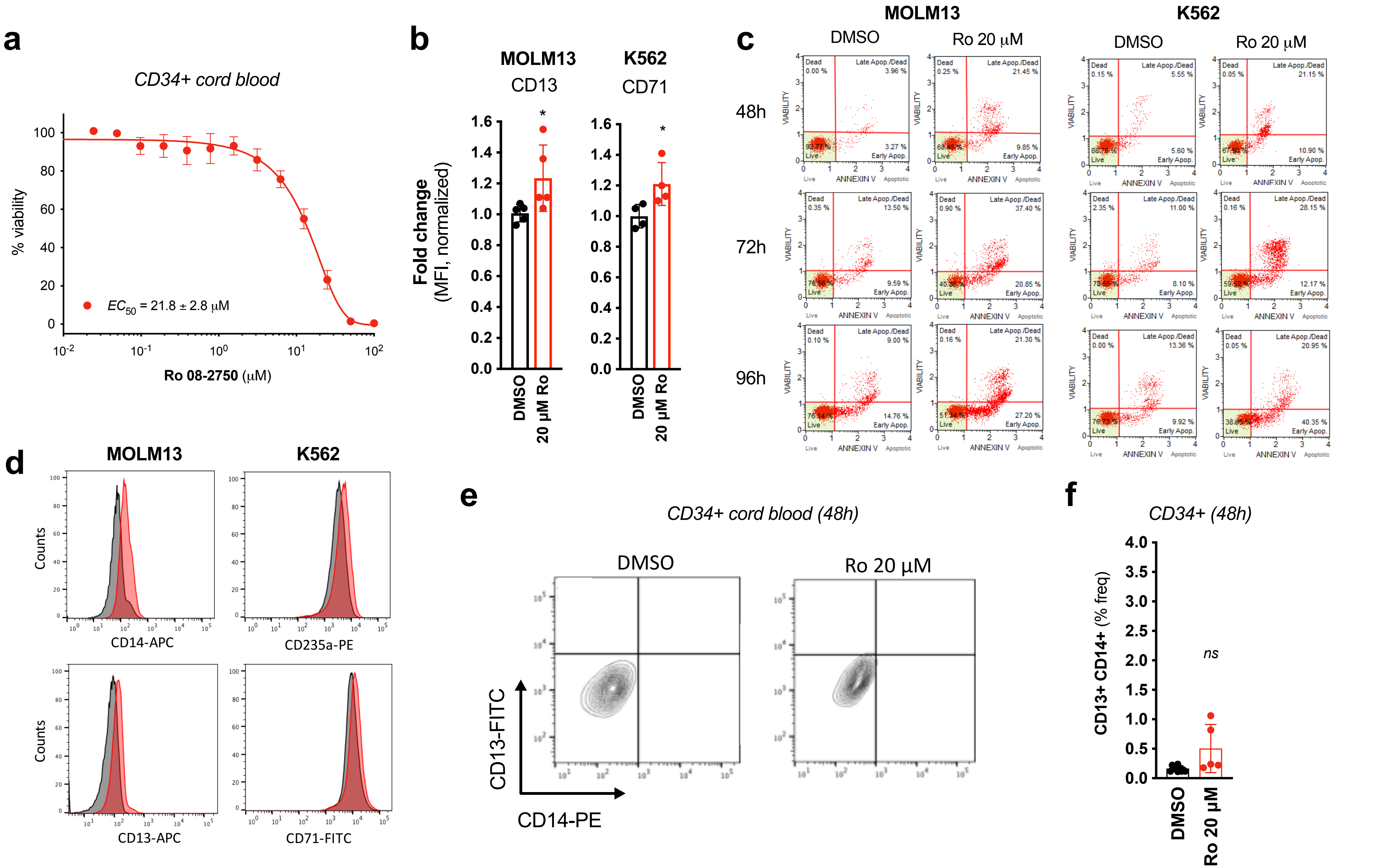

(a) Cytotoxicity assay (Cell-Titer Glo[TM]) of Ro in CD34+ cord blood cells at 72h. 50% Effective Concentration (EC_{50}) values, average of at least four independent experiments ± s.e.m. are shown. (b) Mean Fluorescence Intensity (MFI) fold changes of CD13 (myeloid marker, MOLM13) and CD71 (erythroid marker, K562) after 48h treatment of leukemia cell lines with DMSO (control, *black bars*) or Ro 20 µM (*red bars*). Data is shown as average (normalized to DMSO control cells) ± s.e.m. of three independent experiments performed in triplicate. Paired *t*-test (DMSO vs Ro treated); *$p<0.05$. (c) Apoptosis plots (from graphs in Fig. 4d) showing Annexin V+ and 7AAD (live/dead staining) in MOLM13 and K562 by MUSE Cell Analyzer in DMSO and Ro 20 µM treatments at 48, 72 and 96h. (d) Representative histograms showing CD14 and CD13 myeloid markers in MOLM13 and erythroid differentiation markers CD235a (Glycophorin-A) and CD71 in K562 after 48h of 20 µM Ro treatment. (e) Representative CD13+CD14+ plots for DMSO *vs* Ro 20 µM treated CD34+ cells after 48h. (f) Percentage of frequency (% freq) of CD13+CD14+ in Ro 20 µM treated CD34+ cells after 48h versus control. Paired *t*-test, *ns*, non-significant. Source data are provided as a Source Data file.

Supplementary Figure 7. Ro effects on global translation and mRNA of MSI2 targets.

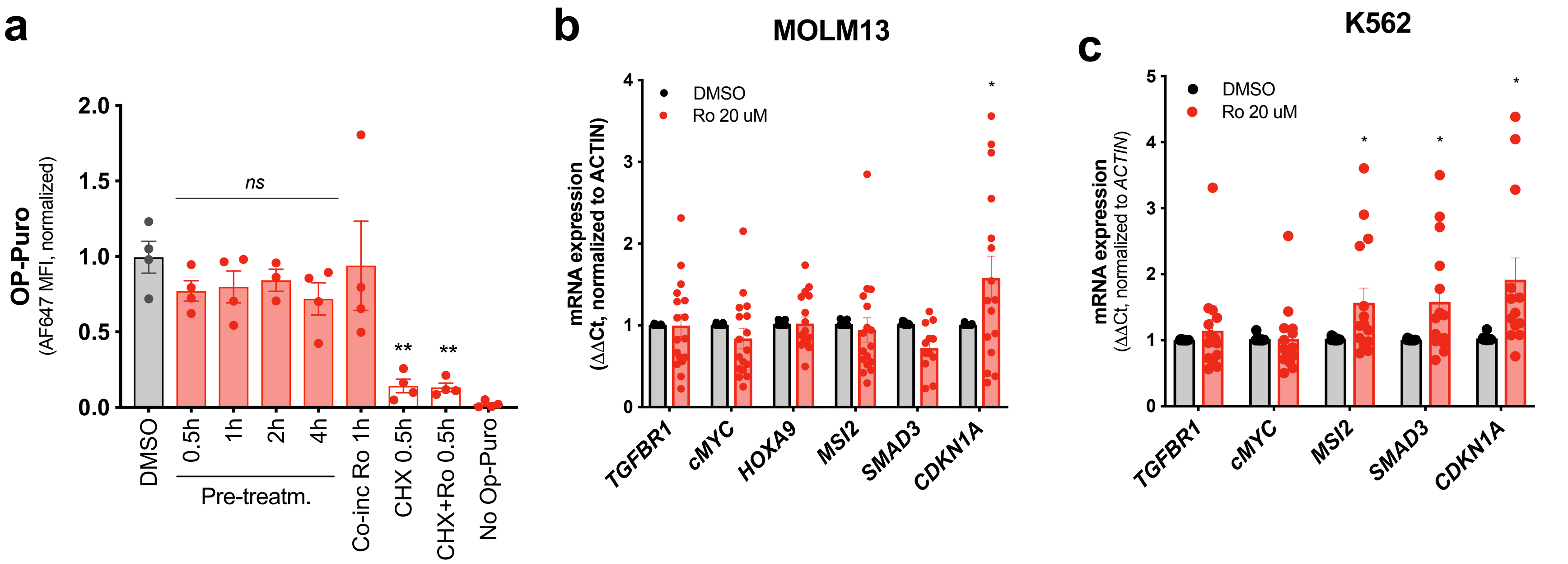

(a) OP-Puromycin incorporation to assess global translation rates in MOLM13 leukemia cells. Results are represented as average of Alexa Fluor 647 (AF647) Mean Fluorescence Intensity (MFI) normalized to DMSO control cells ± s.e.m. of four independent experiments performed in duplicate. Two tailed paired *t*-test (DMSO vs Ro treated); *ns*, non-significant, $^{**}p<0.005$. (b) Expression levels of mRNA targets of MSI2 by qPCR in MOLM13 and (c) K562. Cells were treated for 4h at 20 µM Ro. Results represent the average of at least 12 independent experiments ± s.e.m. *HOXA9* gene is not expressed in K562. Two tailed paired *t*-test (DMSO vs Ro treated); $^{*}p<0.05$. Source data are provided as a Source Data file.

Supplementary Figure 8. No signs of toxicity in vivo after Ro treatment of MLL-AF9 mice.

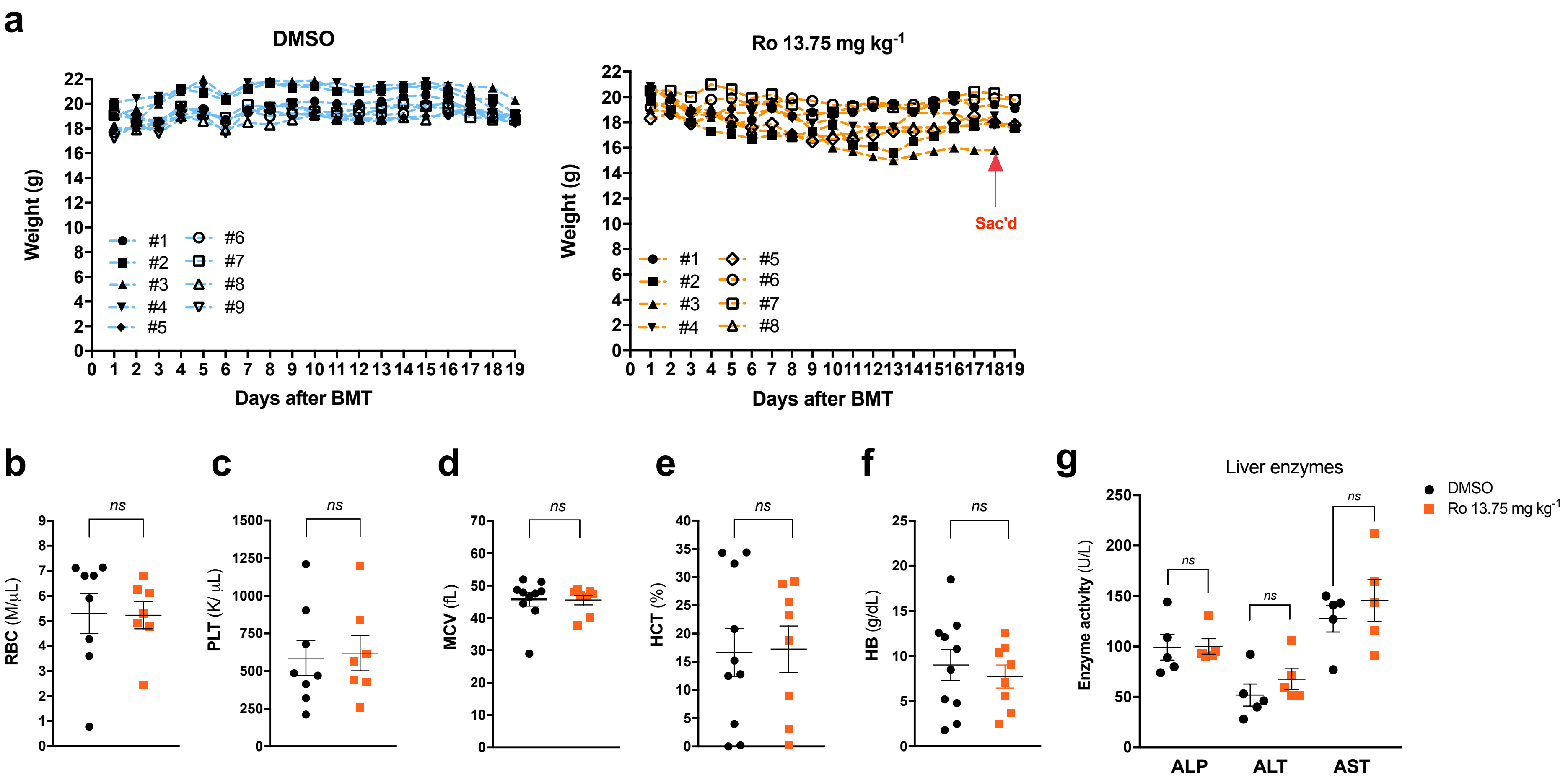

(a) Mice weight in DMSO (*cyan lines*, left panel) and Ro 13.75 mg kg^{-1} (*orange lines*, right panel) groups during the duration of the *in vivo* experiment. (b) Red Blood Cell (RBC) counts (M/ μL), (c) Platelets counts (PLT) counts (K/ μL) (d) Mean Corpuscular Volume (MCV) (fL), (e) Hematocrit (percentage, %), (f) Hemoglobin (g/ dL). Each data point represents an individually treated (DMSO or Ro 13.75 mg kg^{-1}) mouse. Unpaired *t*-test; *ns*, non-significant. (g) Using healthy mice, liver enzyme activity (alkaline phosphatase -ALP-, alanine aminotransferase -ALT- and aspartate aminotransferase -AST-) was assessed after 24 hours of Ro treatment. Each data point represents an individually treated mouse. Unpaired *t*-test; *ns*, non-significant. Source data are provided as a Source Data file.
