# Supplementary material for: Small-molecule targeting of MUSASHI RNA-binding activity in acute myeloid leukemia
Source: Nat Commun. 2019 Jun 19;10:2691. doi: 10.1038/s41467-019-10523-3 (PMC6584500; doi:10.1038/s41467-019-10523-3)
Supplement: Supplementary file 3 — Reporting Summary [file 41467_2019_10523_MOESM3_ESM.pdf]

nature research

Corresponding author(s): Michael G. Kharas

Reporting Summary

Nature Research wishes to improve the reproducibility of the work that we publish. This form provides structure for consistency and transparency in reporting. For further information on Nature Research policies, see Authors & Referees and the Editorial Policy Checklist.

Statistical parameters

When statistical analyses are reported, confirm that the following items are present in the relevant location (e.g. figure legend, table legend, main text, or Methods section).

n/a	Confirmed	
☐	☒	The exact sample size (n) for each experimental group/condition, given as a discrete number and unit of measurement
☐	☒	An indication of whether measurements were taken from distinct samples or whether the same sample was measured repeatedly
☐	☒	The statistical test(s) used AND whether they are one- or two-sided<br>*Only common tests should be described solely by name; describe more complex techniques in the Methods section.*
☐	☒	A description of all covariates tested
☐	☒	A description of any assumptions or corrections, such as tests of normality and adjustment for multiple comparisons
☐	☒	A full description of the statistics including central tendency (e.g. means) or other basic estimates (e.g. regression coefficient) AND variation (e.g. standard deviation) or associated estimates of uncertainty (e.g. confidence intervals)
☐	☒	For null hypothesis testing, the test statistic (e.g. F, t, r) with confidence intervals, effect sizes, degrees of freedom and P value noted<br>*Give P values as exact values whenever suitable.*
☒	☐	For Bayesian analysis, information on the choice of priors and Markov chain Monte Carlo settings
☒	☐	For hierarchical and complex designs, identification of the appropriate level for tests and full reporting of outcomes
☐	☒	Estimates of effect sizes (e.g. Cohen's d, Pearson's r), indicating how they were calculated
☐	☒	Clearly defined error bars<br>*State explicitly what error bars represent (e.g. SD, SE, CI)*

Our web collection on statistics for biologists may be useful.

Software and code

Policy information about availability of computer code

Data collection	All software used for data collection is commercially available and stated in the Methods section (with detailed information of version used). To remark, for FACS, BD FACS LSRII or Fortessa equipment were used, for bulk RNA-seq:Illumina HiSeq platform.
Data analysis	Flowjo 10.4.2 was used to analyze FACS data. Gene Set Enrichment Analysis (GSEA): Gene set enrichment analysis v3.0. was used.<br>All other statistics: GraphPad Prism 7 and 8 software and Microsoft Excel 2016 for Mac. For computational analysis, all Schrödinger project files, YANK simulation inputs and analysis scripts have been made publicly available (https://github.com/choderalab/musashi). All other software used for data analysis is detailed in the Methods section (with detailed information of version used).

For manuscripts utilizing custom algorithms or software that are central to the research but not yet described in published literature, software must be made available to editors/reviewers upon request. We strongly encourage code deposition in a community repository (e.g. GitHub). See the Nature Research guidelines for submitting code & software for further information.

Data

Policy information about availability of data

All manuscripts must include a data availability statement. This statement should provide the following information, where applicable:

- Accession codes, unique identifiers, or web links for publicly available datasets
- A list of figures that have associated raw data
- A description of any restrictions on data availability

Accession codes, unique identifiers or web links are available throughout the manuscript or Methods section. The RRM1 (MSI2) crystal structure has been deposited in RCSB PDB under accession code 6DBP. RNA-seq data has been deposited to GSE114320 and made available (https://www.ncbi.nlm.nih.gov/geo/query/acc.cgi?acc=GSE114320) to all scientific community.

Field-specific reporting

Please select the best fit for your research. If you are not sure, read the appropriate sections before making your selection.

- [x] Life sciences
- [ ] Behavioural & social sciences
- [ ] Ecological, evolutionary & environmental sciences

For a reference copy of the document with all sections, see nature.com/authors/policies/ReportingSummary-flat.pdf

Life sciences study design

All studies must disclose on these points even when the disclosure is negative.

Sample size	For the in vivo experiments we aimed for a number of at least 5 animals per group to allow basic statistical inference. The lowest number of mice was used in the liver enzymes comparison (DMSO vs Ro treated) in Extended Data Figure 8 with 5 versus 5 not-transplanted mice.
Data exclusions	No data were excluded from the analysis with the exception of one mouse from Ro 08-2750 treated arm that was sacrificed one day prior to the termination of the experiment.
Replication	All attempts at replication were successful. Note that for biochemical assays, buffers, pH ranges stated and salt concentrations are crucial for data reproducibility as well as purity of the samples (>90-95% desirable).
Randomization	We allocated recipient mice into different group randomly in transplant and in vivo treatment experiments.
Blinding	No experiments were blinded.

Reporting for specific materials, systems and methods

Materials & experimental systems

n/a	Involved in the study
[ ]	[x] Unique biological materials
[ ]	[x] Antibodies
[ ]	[x] Eukaryotic cell lines
[ ]	[ ] Palaeontology
[ ]	[x] Animals and other organisms
[ ]	[ ] Human research participants

Methods

n/a	Involved in the study
[ ]	[ ] ChIP-seq
[ ]	[x] Flow cytometry
[ ]	[ ] MRI-based neuroimaging

Unique biological materials

Policy information about availability of materials

Obtaining unique materials	Unique materials such as pGEX6P-3-FLAG-MSI2 and RRM1 constructs (including single mutants) are available upon request.

Antibodies

Antibodies used	For FACS mouse cells characterization, anti-CD11b (Mac1)-PE (clone M1/70, #101208, BioLegend), anti-Ly-6G (Gr1)-APC (clone RB6-8C5, #17-5931-82, eBioscience), and anti-CD117 (c-Kit)-APC-Cy7 (clone 2B8, #105826, BioLegend). For the human cell lines

differentiation, we used two panels: (1) anti-CD14-PE (clone M5E2, #555398, BD Pharmingen), anti-CD13-APC (clone TUK1, #MHCD1305, Life Technologies); (2) anti-CD71-FITC (clone CY1G4, #334104, BioLegend), anti-CD235a (Glycophorin A)-PE (clone YTH89.1, #MA5-17700, Invitrogen). For CD34+ differentiation, anti-CD13FITC (clone TUK1, #MHCD1301, Life Technologies and anti-CD14-PE (same as for human cell lines) were used. For intracellular flow cytometry we used anti-cMYC (#5605, Cell Signaling Technology) and donkey anti-rabbit Alexa Fluor 568 (#A10042, Invitrogen) or goat anti-rabbit Alexa Fluor 647 (#A21245, Invitrogen).
For immunoblotting analysis the following antibodies were used: TG[illegible]R1 (ab31013, Abcam, 1:750 dilution), SMAD3 (9523S, Cell Signaling Technology, 1:750 dilution), HOXA9 (07-178, Millipore, for drug dose-dependent experiments and ab140631, Abcam; 1:1,000 dilution for time-course experiments), c-MYC (5605, Cell Signaling Technology; 1:1,000 dilution), P21 (2947S, Cell Signaling Technology, 1:750 dilution), MSI2 (ab76148, Abcam; 1:2,000 dilution) and [illegible]-ACTIN-HRP conjugated (A3854, Sigma-Aldrich; 1:20,000 dilution).
For RNA-IP experiments, a mouse anti-FLAG (clone M2, #F1804 from Sigma-Aldrich) was used.
For O-propargyl-puromycin experiments by FACS, the aforementioned goat anti-rabbit Alexa Fluor 647 secondary antibody (#A21245, Invitrogen) was used.

Validation

Each antibody and clone was initially tested in the laboratory by titration according to manufacturer's instructions with positive and negative cells and signal measurements in Fortesa and LSRII instruments. Relevant antibody profiles for flow cytometry can be found in the commercial source website by searching the above indicated reference.

Eukaryotic cell lines

Policy information about cell lines

Cell line source(s)

K562 (ATCC® CCL-243) and MOLM13 (#ACC 554) lines were purchased from American Type Culture Collection (ATCC) and Deutsche Sammlung von Mikroorganismen und Zellkulturen (DSMZ), respectively.

Authentication

Both cell lines were authenticated and mycoplasma tested by Genetica DNA Lab - Cell Line Testing (www.celllineauthentication.com).

Mycoplasma contamination

Beside Genetica DNA Lab unique test, cells were routinely tested and confirmed negative for mycoplasma in house by using a Mycoplasma Test from Lonza Biosciences (#LT07-218) according to manufacturer's instructions.

Commonly misidentified lines
(See ICLAC register)

n/a

Palaeontology

Specimen provenance

Provide provenance information for specimens and describe permits that were obtained for the work (including the name of the issuing authority, the date of issue, and any identifying information).

Specimen deposition

Indicate where the specimens have been deposited to permit free access by other researchers.

Dating methods

If new dates are provided, describe how they were obtained (e.g. collection, storage, sample pretreatment and measurement), where they were obtained (i.e. lab name), the calibration program and the protocol for quality assurance OR state that no new dates are provided.

☐ Tick this box to confirm that the raw and calibrated dates are available in the paper or in Supplementary Information.

Animals and other organisms

Policy information about studies involving animals; ARRIVE guidelines recommended for reporting animal research

Laboratory animals

Only 10-12 week old female C57BL/6 were used for in vivo experiments (sub-lethally irradiated at 475 cGy before bone marrow transplantation).

Wild animals

n/a

Field-collected samples

n/a

Human research participants

Policy information about studies involving human research participants

Population characteristics

AML patient cells were used and the characteristics of each patient are detailed in Supplementary Table 2.

Recruitment

AML patient cells used in Colony Forming Assays were obtained through Memorial Sloan Kettering Tumor Bank (IRB #18-272)

ChIP-seq

Data deposition

☐ Confirm that both raw and final processed data have been deposited in a public database such as GEO.

☐ Confirm that you have deposited or provided access to graph files (e.g. BED files) for the called peaks.

Data access links<br>*May remain private before publication.*	*For "Initial submission" or "Revised version" documents, provide reviewer access links. For your "Final submission" document, provide a link to the deposited data.*
Files in database submission	*Provide a list of all files available in the database submission.*
Genome browser session<br>(e.g. UCSC)	*Provide a link to an anonymized genome browser session for "Initial submission" and "Revised version" documents only, to enable peer review. Write "no longer applicable" for "Final submission" documents.*

Methodology

Replicates	*Describe the experimental replicates, specifying number, type and replicate agreement.*
Sequencing depth	*Describe the sequencing depth for each experiment, providing the total number of reads, uniquely mapped reads, length of reads and whether they were paired- or single-end.*
Antibodies	*Describe the antibodies used for the ChIP-seq experiments; as applicable, provide supplier name, catalog number, clone name, and lot number.*
Peak calling parameters	*Specify the command line program and parameters used for read mapping and peak calling, including the ChIP, control and index files used.*
Data quality	*Describe the methods used to ensure data quality in full detail, including how many peaks are at FDR 5% and above 5-fold enrichment.*
Software	*Describe the software used to collect and analyze the ChIP-seq data. For custom code that has been deposited into a community repository, provide accession details.*

Flow Cytometry

Plots

Confirm that:

☒ The axis labels state the marker and fluorochrome used (e.g. CD4-FITC).

☒ The axis scales are clearly visible. Include numbers along axes only for bottom left plot of group (a 'group' is an analysis of identical markers).

☒ All plots are contour plots with outliers or pseudocolor plots.

☒ A numerical value for number of cells or percentage (with statistics) is provided.

Methodology

Sample preparation	Age-matched mice were euthanasia by carbon dioxide. Bones from hind legs, forelegs and/or vertebrae were isolated. Bone marrow (BM) was extracted by crushing the bones using mortar and pestle in RPMI + 2% FBS. The suspension was filtered through a 70 µm mesh. Spleen cells were obtained by crushing 1/3 of the spleen volume on a 70 µm mesh. All samples were centrifuged for 5 min at 1,500 rpm. Viable cell numbers were determined using the Trypan blue staining. BM or spleen samples was stained with different cocktails containing antibodies against specific cell surface marker(see detail in method part). Samples were washed, resuspended and subjected to FACS analysis.
Instrument	LSRII and Fortessa Flow cytometers from BD Bioscience were used for FACS data collection. Aria Flow cell sorter from BD Biosciences was used for cell sorting.
Software	FlowJo Software (version 10.4.2) was used for FACS data analysis.
Cell population abundance	The purity of sorted cells were detected via flow cytometer after sorting and samples with purity higher than 95% were used.
Gating strategy	Gating strategies are provided in the Supplementary information file.

☒ Tick this box to confirm that a figure exemplifying the gating strategy is provided in the Supplementary Information.

Magnetic resonance imaging

Experimental design

Design type	*Indicate task or resting state; event-related or block design.*
Design specifications	*Specify the number of blocks, trials or experimental units per session and/or subject, and specify the length of each trial or block (if trials are blocked) and interval between trials.*
Behavioral performance measures	*State number and/or type of variables recorded (e.g. correct button press, response time) and what statistics were used to establish that the subjects were performing the task as expected (e.g. mean, range, and/or standard deviation across subjects).*

Acquisition

Imaging type(s)	*Specify: functional, structural, diffusion, perfusion.*
Field strength	*Specify in Tesla*
Sequence & imaging parameters	*Specify the pulse sequence type (gradient echo, spin echo, etc.), imaging type (EPI, spiral, etc.), field of view, matrix size, slice thickness, orientation and TE/TR/flip angle.*
Area of acquisition	*State whether a whole brain scan was used OR define the area of acquisition, describing how the region was determined.*

Diffusion MRI ☐ Used ☐ Not used

Preprocessing

Preprocessing software	*Provide detail on software version and revision number and on specific parameters (model/functions, brain extraction, segmentation, smoothing kernel size, etc.).*
Normalization	*If data were normalized/standardized, describe the approach(es): specify linear or non-linear and define image types used for transformation OR indicate that data were not normalized and explain rationale for lack of normalization.*
Normalization template	*Describe the template used for normalization/transformation, specifying subject space or group standardized space (e.g. original Talairach, MNI305, ICBM152) OR indicate that the data were not normalized.*
Noise and artifact removal	*Describe your procedure(s) for artifact and structured noise removal, specifying motion parameters, tissue signals and physiological signals (heart rate, respiration).*
Volume censoring	*Define your software and/or method and criteria for volume censoring, and state the extent of such censoring.*

Statistical modeling & inference

Model type and settings	*Specify type (mass univariate, multivariate, RSA, predictive, etc.) and describe essential details of the model at the first and second levels (e.g. fixed, random or mixed effects; drift or auto-correlation).*
Effect(s) tested	*Define precise effect in terms of the task or stimulus conditions instead of psychological concepts and indicate whether ANOVA or factorial designs were used.*

Specify type of analysis: ☐ Whole brain ☐ ROI-based ☐ Both

Statistic type for inference (See Eklund et al. 2016)	*Specify voxel-wise or cluster-wise and report all relevant parameters for cluster-wise methods.*
Correction	*Describe the type of correction and how it is obtained for multiple comparisons (e.g. FWE, FDR, permutation or Monte Carlo).*

Models & analysis

n/a	Involved in the study
☐	☐ Functional and/or effective connectivity
☐	☐ Graph analysis
☐	☐ Multivariate modeling or predictive analysis

Functional and/or effective connectivity	*Report the measures of dependence used and the model details (e.g. Pearson correlation, partial correlation, mutual information).*
Graph analysis	*Report the dependent variable and connectivity measure, specifying weighted graph or binarized graph, subject- or group-level, and the global and/or node summaries used (e.g. clustering coefficient, efficiency, etc.).*

Multivariate modeling and predictive analysis

Specify independent variables, features extraction and dimension reduction, model, training and evaluation metrics.
