# Supplementary material for: Small-molecule targeting of MUSASHI RNA-binding activity in acute myeloid leukemia
Source: Nat Commun. 2019 Jun 19;10:2691. doi: 10.1038/s41467-019-10523-3 (PMC6584500; doi:10.1038/s41467-019-10523-3)
Supplement: Supplementary file 4 — Description of Additional Supplementary Files [file 41467_2019_10523_MOESM4_ESM.docx]

**Description of Additional Supplementary Files**

File Name: Supplementary Data 1
Description: Ro-NGF analogue series and their affinity binding to NGF and Bovine Serum Albumin (BSA). Chemical structures, binding to NGF and BSA for Ro-NGF compound analogues.

File Name: Supplementary Data 2

Description: AML patient characteristics. Description of cell type, number, blast, age, gender and genetic background of their myeloid leukemia are detailed.

File Name: Supplementary Data 3

Description: Gene expression changes in MOLM13 from RNA-sequencing data after Ro treatment.

File Name: Supplementary Data 4

Description: Gene expression changes in K562 from RNA-sequencing data after Ro treatment.

File Name: Supplementary Data 5

Description: Rank list from shRNAs in AML and CML-BC cell lines from previous data set in Kharas *et al.*

File Name: Supplementary Data 6

Description: MOLM13 Gene Set Enrichment Analysis (GSEAs) positively enriched (upregulated)

(*p* value< 0.05, FDR<0.2)

File Name: Supplementary Data 7

Description: MOLM13 GSEAs negatively enriched (downregulated)

(*p* value< 0.05, FDR<0.2)

File Name: Supplementary Data 8

Description: K562 GSEAs positively enriched (upregulated)

(*p* value< 0.05, FDR<0.2)

File Name: Supplementary Data 9

Description: K562 GSEAs negatively enriched (downregulated)

(*p* value< 0.05, FDR<0.2)

File Name: Supplementary Data 10
Description: MSI2-shRNA in CML-BC and AML cell lines; GSEAs positively enriched (upregulated).

(*p* value< 0.05, FDR<0.2)

File Name: Supplementary Data 11

Description: MSI2-shRNA in CML-BC and AML cell lines; GSEAs negatively enriched (downregulated).

(*p* value< 0.05, FDR<0.2)

File Name: Supplementary Data 12

Description: Overlap of GSEAs. In red are MSI related pathways.
