# Supplementary material for: Small-molecule targeting of MUSASHI RNA-binding activity in acute myeloid leukemia
Source: Nat Commun. 2019 Jun 19;10:2691. doi: 10.1038/s41467-019-10523-3 (PMC6584500; doi:10.1038/s41467-019-10523-3)
Supplement: Supplementary file 2 — Peer Review File [file 41467_2019_10523_MOESM2_ESM.pdf]

Reviewers' comments:

Reviewer #1 (Remarks to the Author):

In this manuscript, Minuesa et al. describe the development of a small molecule, Ro 08-2750 (Ro), that inhibits the binding of MUSASHI2 (MSI2) to RNA. MSI2 has previously been shown to be required for BCR-ABL leukemia. Here the authors show that MSI inhibition by Ro induces differentiation and apoptosis in two types of leukemia, CML and AML, expressing chimeric fusions BCR-ABL or MLL-AF9, respectively. The identification of Ro is of high significance as it provides proof-of-principle for targeting MSI2 in the treatment of leukemias, and presumably other cancers expressing MUSASHI.
In general, the manuscript is clearly written and the results are compelling. The development of specific inhibitors for RNA-binding proteins is challenging. The authors here demonstrate that Ro directly binds to the RRM1 of MSI2 and competes for RNA binding. They also show that Ro induce apoptosis and differentiation of mouse and human leukemia cells, and correlative function in reducing leukemia burden in mice. This study opens the door for evaluating the efficacy of such inhibitors to other cancer types.

The following four specific comments would be required to strengthening the results here reported:

1. Ro shows specificity for MUSASHI and with compelling lethal effect in leukemic cells. Considering that Ro function is observed at micromolar concentrations, it may not be a clinically relevant compound. Ro is a valuable proof-of-principle molecule, and future efforts may develop Ro analogs with nano- or pico-molar efficacy. Have the authors tested the PK or PD of Ro?

2. It is surprising that the mouse experiments do not report the leukemia latency of MLL-AF9 transplanted leukemia cells treated with DMSO or Ro, using a Kaplan-Meier survival curve. Is the leukemia latency in the Ro group extended?. In case the latency is not extended, this should be shown and described in the manuscript. It is stated in the Discussion section that "Despite the challenges for in vivo administration, we reduced the disease burden in an aggressive MLL-AF9 leukemia model and decreased c-MYC levels without overt toxicity". However, this is not described in the Results section. What were the challenges?

3. In Figure 3C, the morphology of differentiated cells is shown at 5μM and 10μM with illustrations, but it is not quantified. This point would be most compelling if a differential counting of myeloid cells is quantified for each condition and shown next to the illustration. Similar quantification should be shown in Figure 4C for human cells.

4. The potential toxicity of Ro in human CD34+ cells, as shown in a CFU assay, is incomplete. Toxicity would be best evaluated by estimating the viability and differentiation of CD34+ human hematopoietic cells in a dose response assay of Ro, including the 1 – 40 μM cc points.

Reviewer #2 (Remarks to the Author):

In this manuscript, Minuesa et al, employed a small molecule screen for inhibitors of the Musashi family proteins (MSI1 and MSI2) and identified the molecule Ro as a selective inhibitor of MSI1/MSI2 RNA-binding activity. Using biochemical approaches and leukemia models, the authors demonstrate the efficacy of using Ro in myeloid leukemias. Genetic studies on this small-molecule demonstrate an overlap of RNA targets upon pharmacological inhibition when compared to knockdown of MSI2 in myeloid leukemia. Overall, this is a very interesting study and presents an approach to target RBPs in cancer, which has been previously been extremely difficult. Furthermore, Musashi proteins are highly deregulated in several other types of cancers and therefore this small-molecule, Ro represents a

therapeutic approach for targeting MS1/2-dependent cancers. However, there are a few comments to be addressed by the authors to further improve the quality of this manuscript.

Major points
• The authors demonstrate the specificity of Ro interacting the Musashi proteins and lower binding with SYNCRIP. However, this is just one example, can the authors also demonstrate it with additional proteins which also have highly conserved RRM1s?

• Previously the authors identified SYNCRIP as a direct interactor of MSI2 in maintaining myeloid leukemia survival (Vu et al., 2017). Though Ro does not demonstrate a strong affinity for the RRM on SYNCRIP, however does Ro perturb the binding to MSI2?

• In Figure 3e, the authors overexpressed MSI2 to rescue the effects of Ro. However, as shown in Figure 3f, overexpression of MSI2 dramatically increases translation of c-MYC, HOXA9, etc…) compared to empty vector and therefore represents an unfair assessment of these rescue experiments. For a more direction comparison, can the authors perform rescue experiments by overexpressing a precise mutation of MSI2 that they identified in this paper that renders Ro ineffective?

• In Figure 6, the authors demonstrate a strong efficacy of Ro using mouse AML models. Is there a survival benefit of leukemic mice treated with Ro in Figure 6a or 6d experiemnts?

Minor points
• The authors demonstrate that Ro actively decreases HOXA9 protein expression but do the authors also see HOXA9 target genes downregulated transcriptionally?

• In the in vivo experiments, the authors choose a 13.75 mg/kg dose, which seem to be much better than the in vitro effects. Is there a rationale for choosing 13.75 mg/kg and have the authors tested higher doses for a more stronger effect in vivo?

• In Figure 6d, does c-kit expression also decreased in this treatment condition as shown in Figure 6b

• Labels are missing for the flow cytometry plots for Supplementary 6a.

• In Supplementary Figure 7c, there seems to be a significant increase in MSI2 mRNA expression (as well as SMAD3 and CDKN1A) upon Ro treatment. Can the authors comment? Does MSI2 knockdown also affect MSI2 transcripts level?

Reviewer #3 (Remarks to the Author):

This very interesting manuscript reports the discovery as well as extensive cellular and in vivo characterization of a small molecule that binds to the Musashi RRM domains and inhibits its function in regulating translation of proto-oncogenic mRNAs.

Targeting undruggable oncogenic proteins (c-myc, k-Ras, STAT3) by downregulating their mRNAs is a sound idea, and after all the foundation of many antisense programs that failed because of the platform (penetration of solid tumors etc). Thus, targeting these same mRNAs with small molecules is appealing, but mRNAs have relatively little structure and are coated with hnRNPs to keep them that way. The option chosen by the authors is to target one of the proteins, many of which are RRM or KH domains that regulate these mRNAs, as has been known for 30 years. The challenge is the flat RNA recognition surfaces especially of RRMs, which provide a classical protein-protein-like inhibition challenge.

Here the authors report the discovery, using a straightforward RNA displacement assay, of a small molecule that binds Musashi-2 RRM at the RNP-1 and RNP-2 sequences that define the RRM, inhibits binding of mRNAs to the protein and, especially, have various very attractive properties in cellular and in vivo models of leukemia, including inhibition of c-Myc and reduced disease burden in mouse models.

This is impressive and very interesting work, with one significant caveat; the molecule has low uM binding affinity (2-12 uM depending on the assay), which is not surprising given the molecule and the surface. Many inhibitors of protein-protein interactions have been reported with low uM binding in high profile journals, but never seem to move beyond academia and into the clinic, I suspect because it becomes difficult to raise potency and specificity. In addition, the molecule also seems to have delivery problems and requires injection, which is not a good characteristic of course for a small molecule.

Still, as a proof of principle of what can be achieved by targeting Musashi, this work is of very high interest and quality, and I would very much like to see it published, and I say this with enthusiasm.

However, before I can fully recommend publication, there are two issues that I would like to see addressed.
1. How specific is this molecule? There are hundreds of RRMs in humans, most look the same, most have very similar RNP-1 and RNP-2 sequences. How many more are inhibited? If Ro really binds to hydrophobic/aromatic residues in RNP-1 and RNP-2, this molecule might bind to many RRMs or some of them. I would like to see more specificity controls, beyond SYNCRIP, namely a panel of RRMs which the authors can pick from the literature, 4-5 soluble RRMs, and show that the molecule does not bind.
2. The identification of the binding site on the RRM surface is not fully credible. Without direct binding data, the authors use docking, which is replete with limitations with these featureless binding surfaces. The authors could use NMR chemical shift changes to map the binding site (I seem to remember NMR studies of Musashi from a Japanese group, about 15 years ago); this would not take long. Alternatively or in addition, the authors could show that the triple mutant introduced on the RRM surface does not disrupt the protein fold.

With regards to point 2, I will add that I think the authors are right, based on the data presented. However, I also hope (for their future progress) that they are wrong and the molecule binds instead closer to one of the loops at the bottom of the RRM, the 2-3 loop especially, that are so important for RNA binding and that sort of fold against the b-sheet sometimes to provide much more inviting binding pockets.

These points are somewhat important in light of the very modest SAR (2 molecules); I understand it is difficult to do small molecule work in Academia, so this sort of validation from the point of view of the target is more important.

Once these technical points are addressed, then I would very strongly support publication.

Reviewers' comments:

Reviewer #1 (Remarks to the Author):

In this manuscript, Minuesa et al. describe the development of a small molecule, Ro 08-2750 (Ro), that inhibits the binding of MUSASHI2 (MSI2) to RNA. MSI2 has previously been shown to be required for BCR-ABL leukemia. Here the authors show that MSI inhibition by Ro induces differentiation and apoptosis in two types of leukemia, CML and AML, expressing chimeric fusions BCR-ABL or MLL-AF9, respectively. The identification of Ro is of high significance as it provides proof-of-principle for targeting MSI2 in the treatment of leukemias, and presumably other cancers expressing MUSASHI.

In general, the manuscript is clearly written and the results are compelling. The development of specific inhibitors for RNA-binding proteins is challenging. The authors here demonstrate that Ro directly binds to the RRM1 of MSI2 and competes for RNA binding. They also show that Ro induce apoptosis and differentiation of mouse and human leukemia cells, and correlative function in reducing leukemia burden in mice. This study opens the door for evaluating the efficacy of such inhibitors to other cancer types.

The following four specific comments would be required to strengthening the results here reported:

1. Ro shows specificity for MUSASHI and with compelling lethal effect in leukemic cells. Considering that Ro function is observed at micromolar concentrations, it may not be a clinically relevant compound. Ro is a valuable proof-of-principle molecule, and future efforts may develop Ro analogs with nano- or pico-molar efficacy. Have the authors tested the PK or PD of Ro?

We have not evaluated the PK of Ro but using MYC as a pharmacodynamic (PD) marker in vivo we found reduced abundance as early as 4hrs and 12hrs post treatment (see the original Figure 6a and 6b).

2. It is surprising that the mouse experiments do not report the leukemia latency of MLL-AF9 transplanted leukemia cells treated with DMSO or Ro, using a Kaplan-Meier survival curve. Is the leukemia latency in the Ro group extended?. In case the latency is not extended, this should be shown and described in the manuscript. It is stated in the Discussion section that “Despite the challenges for in vivo administration, we reduced the disease burden in an aggressive MLL-AF9 leukemia model and decreased c-MYC levels without overt toxicity”. However, this is not described in the Results section. What were the challenges?

Unfortunately, the leukemia latency was not extended in this very aggressive leukemia model. Due to poor solubility and considering the top concentration in DMSO we can achieve is 20 mM, we could only reach the 13.75 mg/kg reported in the paper. Also, DMSO dosing (50 µL x injection) limits our ability to dose animals higher.

3. In Figure 3C, the morphology of differentiated cells is shown at 5µM and 10µM with illustrations, but it is not quantified. This point would be most compelling if a differential counting of myeloid cells is quantified for each condition and shown next to the illustration. Similar quantification should be shown in Figure 4C for human cells.

We believe that flow cytometry is a more quantitative and less biased way to measure differentiation than morphology in cytospins. Thus, we only provided the cytospins as a visual representative images and depiction of differentiation.

4. The potential toxicity of Ro in human CD34+ cells, as shown in a CFU assay, is incomplete. Toxicity would be best evaluated by estimating the viability and differentiation of CD34+ human hematopoietic cells in a dose response assay of Ro, including the 1 – 40 μM cc points.

We now have added a cell viability assay providing an EC_{50} of 21.8 μM and we observed no significant change in differentiation (**Extended Data Fig. 6d-f**). This 2-fold difference in differential toxicity in human AML cell lines is consistent with the other in vitro data we have already provided that include colony assays utilizing immature hematopoietic stem and progenitor cell populations from mouse and human origin. More importantly, in our in vivo experiments where we observed therapeutic efficacy there was no difference in platelets, RBCs, other blood parameters, weight loss, or an increase in liver enzymes. These data suggest a differential effect of Ro on leukemia cells compared to normal cells and in vivo therapeutic index. See lines **263-267** (**Results** section).

Reviewer #2 (Remarks to the Author):

In this manuscript, Minuesa et al, employed a small molecule screen for inhibitors of the Musashi family proteins (MSI1 and MSI2) and identified the molecule Ro as a selective inhibitor of MSI1/MSI2 RNA-binding activity. Using biochemical approaches and leukemia models, the authors demonstrate the efficacy of using Ro in myeloid leukemias. Genetic studies on this small-molecule demonstrate an overlap of RNA targets upon pharmacological inhibition when compared to knockdown of MSI2 in myeloid leukemia. Overall, this is a very interesting study and presents an approach to target RBPs in cancer, which has been previously been extremely difficult. Furthermore, Musashi proteins are highly deregulated in several other types of cancers and therefore this small-molecule, Ro represents a therapeutic approach for targeting MS1/2-dependent cancers. However, there are a few comments to be addressed by the authors to further improve the quality of this manuscript.

Major points

• The authors demonstrate the specificity of Ro interacting the Musashi proteins and lower binding with SYNCRIP. However, this is just one example, can the authors also demonstrate it with additional proteins **which also have highly conserved RRM1s**?

We now have added a panel including five RBPs with conserved RRM1s that demonstrate significantly reduced or no direct binding to Ro as assessed through the MST assay (i.e. MSI K_D = 12.3±0.5 μM, SYNCRIP K_D = 236±167 μM, SFSR2 K_D = 190±60 μM, HUR, RBMX, TIA-1 and ALBUMIN K_D ≥ 500 μM), (new Figure 1d). This adds an important piece of information to claim that, despite its micromolar interaction, Ro 08-2750 demonstrates specificity to MSI2 compared to a set of RBPs with closely conserved RRMs. See **lines 210-213** (Results section) and **458-460** (Discussion section).

• Previously the authors identified SYNCRIP as a direct interactor of MSI2 in maintaining myeloid leukemia survival (Vu et al., 2017). Though Ro does not demonstrate a strong affinity for the RRM on SYNCRIP, however does Ro perturb the binding to MSI2?

Previously we found that SYNCRIP interacts with MSI2 via shared targets but not directly as RNAse disrupted their association. Thus, it is not clear to us the importance or relevance for Ro's ability to perturb MSI2's interaction with SYCNRIP on target RNAs. We believe that future studies could address how Ro alters MSI2's direct and indirect protein interactions with its many protein-protein and protein-RNA-protein interactors.

• In Figure 3e, the authors overexpressed MSI2 to rescue the effects of Ro. However, as shown in Figure 3f, overexpression of MSI2 dramatically increases translation of c-MYC, HOXA9, etc…) compared to empty vector and therefore represents an unfair assessment of these rescue experiments. For a more direction comparison,

can the authors perform rescue experiments by overexpressing a precise mutation of MSI2 that they identified in this paper that renders Ro ineffective?

In our original manuscript, we found that MSI2 wildtype overexpression already partially rescues the effect of Ro, we now provide additional data using overexpression of the single mutants (K22A, F66A, F97A or R100A) with decreased Ro binding, we can significantly increase the colony forming ability compared to the MSI2 WT in the presence of 10 μM of Ro). See **Extended Data Fig. 5b** and **5c** and **lines 251-253** (Results section).

• In Figure 6, the authors demonstrate a strong efficacy of Ro using mouse AML models. Is there a survival benefit of leukemic mice treated with Ro in Figure 6a or 6d experiments?

In **Figure 6a** we sacrificed all the mice at a specific time point to assess PD markers. For serial dosing experiments, the leukemia latency was not extended in this very aggressive leukemia model. All mice were sacked at the same time as they were moribund and reached criteria for ending the experiment. We noted in the text that there was no difference in survival latency for the in vivo leukemia experiments (line **344**, **Results** section). We were not able to achieve high enough dosing *in vivo* due to poor solubility in DMSO (stated in lines **335-336** in the **Results**). Nevertheless, we provide a proof of concept that this drug can be used *in vivo* and that it has quantitative efficacy on disease burden.

Minor points

• The authors demonstrate that Ro actively decreases HOXA9 protein expression but do the authors also see HOXA9 target genes downregulated transcriptionally?

We found HOXA9 and MEIS1 target gene sets altered as observed in our pathway GSEA analysis (Supplementary Table 10). We observed an enrichment of HOXA9 containing gene sets (Hess and Wang HOXA9/MEIS1 Target genes) indicating there was an effect of HOXA9 target genes similar to shRNA depletion of MSI2.

TAKEDA_TARGETS_OF_NUP98_HOXA9_FUSION_10D_UP
TAKEDA_TARGETS_OF_NUP98_HOXA9_FUSION_16D_UP
WANG.LGMPHOXA9-MEIS1VSHSC_UP
HESS_TARGETS_OF_HOXA9_AND_MEIS1_UP
DORSAM_HOXA9_TARGETS_UP

• In the in vivo experiments, the authors choose a 13.75 mg/kg dose, which seem to be much better than the in vitro effects. Is there a rationale for choosing 13.75 mg/kg and have the authors tested higher doses for a more stronger effect in vivo?

Due to poor solubility and considering the top concentration in DMSO we can achieve is 20 mM, we could only reach the 13.75 mg/kg reported in the paper. Also, DMSO dosing (50 μL x injection) limits our ability to dose animal higher. These were the considerations for choosing this dose. We have clearly stated that in the Results section (lines **335-336**).

• In Figure 6d, does c-kit expression also decreased in this treatment condition as shown in Figure 6b?

We have now checked for c-Kit expression in the long-term treatment and we observed a modest increase in c-Kit suggesting a possible feedback response. It is not clear if this increase in c-Kit correlates to an actual enrichment of functionally more immature cells. As the mice died with equivalent latency this indicated we are

likely not inhibiting MSI2 at high enough levels to maintain a response. We decided not to include this data as we still do not understand exactly the mechanism behind this result.

• Labels are missing for the flow cytometry plots for Supplementary 6a.

We have now added the labels.

• In Supplementary Figure 7c, there seems to be a significant increase in MSI2 mRNA expression (as well as SMAD3 and CDKN1A) upon Ro treatment. Can the authors comment? Does MSI2 knockdown also affect MSI2 transcripts level?

This is likely a compensation but the effect is modest less than 2-fold and it was only observed in K562 cells. The protein expression of MSI2 was not significantly changed.

Reviewer #3 (Remarks to the Author):

This very interesting manuscript reports the discovery as well as extensive cellular and in vivo characterization of a small molecule that binds to the Musashi RRM domains and inhibits its function in regulating translation of proto-oncogenic mRNAs.

Targeting undruggable oncogenic proteins (c-myc, k-Ras, STAT3) by downregulating their mRNAs is a sound idea, and after all the foundation of many antisense programs that failed because of the platform (penetration of solid tumors etc). Thus, targeting these same mRNAs with small molecules is appealing, but mRNAs have relatively little structure and are coated with hnRNPs to keep them that way. The option chosen by the authors is to target one of the proteins, many of which are RRM or KH domains that regulate these mRNAs, as has been known for 30 years. The challenge is the flat RNA recognition surfaces especially of RRMs, which provide a classical protein-protein-like inhibition challenge.

Here the authors report the discovery, using a straightforward RNA displacement assay, of a small molecule that binds Musashi-2 RRM at the RNP-1 and RNP-2 sequences that define the RRM, inhibits binding of mRNAs to the protein and, especially, have various very attractive properties in cellular and in vivo models of leukemia, including inhibition of c-Myc and reduced disease burden in mouse models.

This is impressive and very interesting work, with one significant caveat; the molecule has low uM binding affinity (2-12 uM depending on the assay), which is not surprising given the molecule and the surface. Many inhibitors of protein-protein interactions have been reported with low uM binding in high profile journals, but never seem to move beyond academia and into the clinic, I suspect because it becomes difficult to raise potency and specificity. In addition, the molecule also seems to have delivery problems and requires injection, which is not a good characteristic of course for a small molecule.

Still, as a proof of principle of what can be achieved by targeting Musashi, this work is of very high interest and quality, and I would very much like to see it published, and I say this with enthusiasm.

However, before I can fully recommend publication, there are two issues that I would like to see addressed.

1. How specific is this molecule? There are hundreds of RRMs in humans, most look the same, most have very similar RNP-1 and RNP-2 sequences. How many more are inhibited? If Ro really binds to hydrophobic/aromatic residues in RNP-1 and RNP-2, this molecule might bind to many RRMs or some of them. I would like to see more specificity controls, beyond SYNCRIP, namely a panel of RRMs which the authors can pick from the literature, 4-5 soluble RRMs, and show that the molecule does not bind.

We now have a panel including five RBPs with conserved RRM1s that demonstrate significantly reduced or no direct binding to Ro assessed through the MST assay (i.e.: MSI K_D = 12.3±0.5 μM, SFSR2 K_D = 190±60 μM, HUR, RBMX, TIA-1, and ALBUMIN $K_D \geq 500$ μM), (new **Fig. 1d**). This adds an important piece of information to claim that, despite its micromolar interaction and his lipophilic character, Ro 08-2750 shows a good degree of specificity towards MSI family of RBPs. See lines **103-106** (**Results** section).

2. The identification of the binding site on the RRM surface is not fully credible. Without direct binding data, the authors use docking, which is replete with limitations with these featureless binding surfaces. The authors could use NMR chemical shift changes to map the binding site (I seem to remember NMR studies of Musashi from a Japanese group, about 15 years ago); this would not take long. Alternatively or in addition, the authors could show that the triple mutant introduced on the RRM surface does not disrupt the protein fold. With regards to point 2, I will add that I think the authors are right, based on the data presented. However, I also hope (for their future progress) that they are wrong and the molecule binds instead closer to one of the loops

at the bottom of the RRM, the 2-3 loop especially, that are so important for RNA binding and that sort of fold against the b-sheet sometimes to provide much more inviting binding pockets.

We now provide NMR studies that further support the proposed docking model. Most importantly, we found significant chemical shifts that fit with the binding surfaces and residues that when mutated no longer bind our drug and have reduced binding activity and reduced effect on the colony assays. For example, we find NMR shifts in residue F97 and F66 which was also critical in our mutation studies. The other shifting residues (M21, V95, G65, G26 and I25) are within the same region consistent with our docking model. See new **Fig. 2e** and **Extended Data Fig.2c** and **2d** and lines **153-158** in the **Results** section.

These points are somewhat important in light of the very modest SAR (2 molecules); I understand it is difficult to do small molecule work in Academia, so this sort of validation from the point of view of the target is more important.

We appreciate that the reviewer understands the challenges of developing a more robust SAR campaign in academia. Hopefully, our work will provide a strong starting point for future studies that explore more molecules with improved PK, binding affinity and activity.

Once these technical points are addressed, then I would very strongly support publication.

We thank the reviewer for their positive assessment of our work and hope that they will appreciate our extensive revisions and new data that we have provide.

REVIEWERS' COMMENTS:

Reviewer #1 (Remarks to the Author):

The report of Ro as an inhibitor of MISASHI is of high interest in the leukemia field, and with potential therapeutic implications. The revised manuscript has addressed my previous concerns.

Reviewer #2 (Remarks to the Author):

the authors have convincingly addressed my comments and included additional experimental evidence supporting their claims. I believe that now the manuscript is fit for publication.

Reviewer #3 (Remarks to the Author):

The authors have addressed my suggestions by providing new binding data to a panel of RRMs, as well as validation of the binding site by NMR. They were very responsive; I am fully supportive of publication.
